# Synthesis and Structure–Activity Studies of β-Barrel Assembly Machine Complex Inhibitor MRL-494

**DOI:** 10.1021/acsinfecdis.2c00459

**Published:** 2022-11-01

**Authors:** Nicola Wade, Charlotte M. J. Wesseling, Paolo Innocenti, Cornelis J. Slingerland, Gregory M. Koningstein, Joen Luirink, Nathaniel I. Martin

**Affiliations:** † Biological Chemistry Group, Institute of Biology Leiden, Leiden University, 2333 BE Leiden, The Netherlands; ‡ Department of Molecular Microbiology, Amsterdam Institute of Molecular and Life Sciences (AIMMS), Vrije Universiteit, 1081 HV Amsterdam, The Netherlands

**Keywords:** antibiotic, BAM complex, BamA inhibitor, MRL-494, synergy

## Abstract

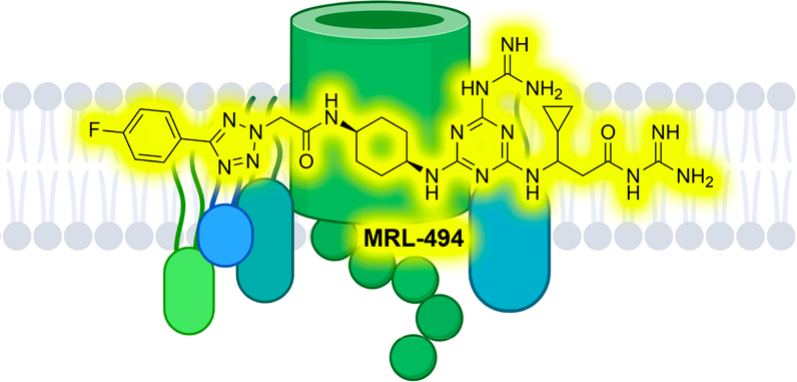

In the hunt for new antibiotics with activity against Gram-negative pathogens, the outer membrane β-barrel assembly machine (BAM) complex has become an increasingly interesting target. The recently reported BAM complex inhibitor, MRL-494, was discovered via a screening campaign for molecules that target the outer membrane. Notably, MRL-494 was reported to be an unintended byproduct generated during the synthesis of an unrelated compound, and as such no synthesis of the compound was disclosed. We here present a convenient and reliable route for the synthesis of MRL-494 that scales well. The antibacterial activity measured for synthesized MRL-494 matches that reported in the literature. Furthermore, MRL-494 was found to exhibit potent synergistic activity with rifampicin against Gram-negative bacteria, including *E. coli*, *K. pneumoniae*, *A. baumannii*, and *P. aeruginosa*. MRL-494 was also found to cause outer membrane disruption and induction of the Rcs stress response pathway. In addition, we undertook a focused structure–activity study specifically aimed at elucidating the roles played by the two guanidine moieties contained within the structure of MRL-494.

Antibiotic resistance is one of the biggest challenges facing modern medicine, with an estimated 1.27 million deaths attributed to bacterial antimicrobial resistance in 2019.^1^ The continued emergence of multi-drug-resistant bacteria, most notably Gram-negative strains, makes clear the need to develop novel therapeutics. In order to effectively counter the growing tide of antibiotic resistance, it is important to identify new bacterial pathways and targets that have not yet been exploited.^2,3^ One such pathway in Gram-negative pathogens is that which governs the production of outer membrane proteins (OMPs), in which the β-barrel assembly machine (BAM) complex plays a crucial role. OMPs are produced in the cytoplasm and are transported via Sec and Sur chaperone proteins to the BAM complex located in the outer membrane (OM), which in turn ensures their correct folding and insertion into the OM (Figure 1).^4−9^ Given the essential nature of OMP production for Gram-negative bacteria, many species have developed stress responses that are activated if problems arise in this pathway.^10,11^ Structurally, the BAM complex is comprised of a β-barrel transmembrane domain (BamA) and four lipoprotein subunits (BamB–E). BamA is connected to the subunits by five polypeptide transport-associated (POTRA) domains.^12,13^ Notably, only BamA and BamD are essential for the activity of the complex. In recent years, growing attention has been paid to the potential for developing compounds capable of inhibiting the activity of the BAM complex as a new avenue for antibiotic discovery. Given that BamA is exposed on the bacterial cell surface, inhibitors that target the BAM complex may not face the same challenges as other antibiotic candidates as relates to their crossing the OM or being ejected by efflux pumps.

**Figure 1:**
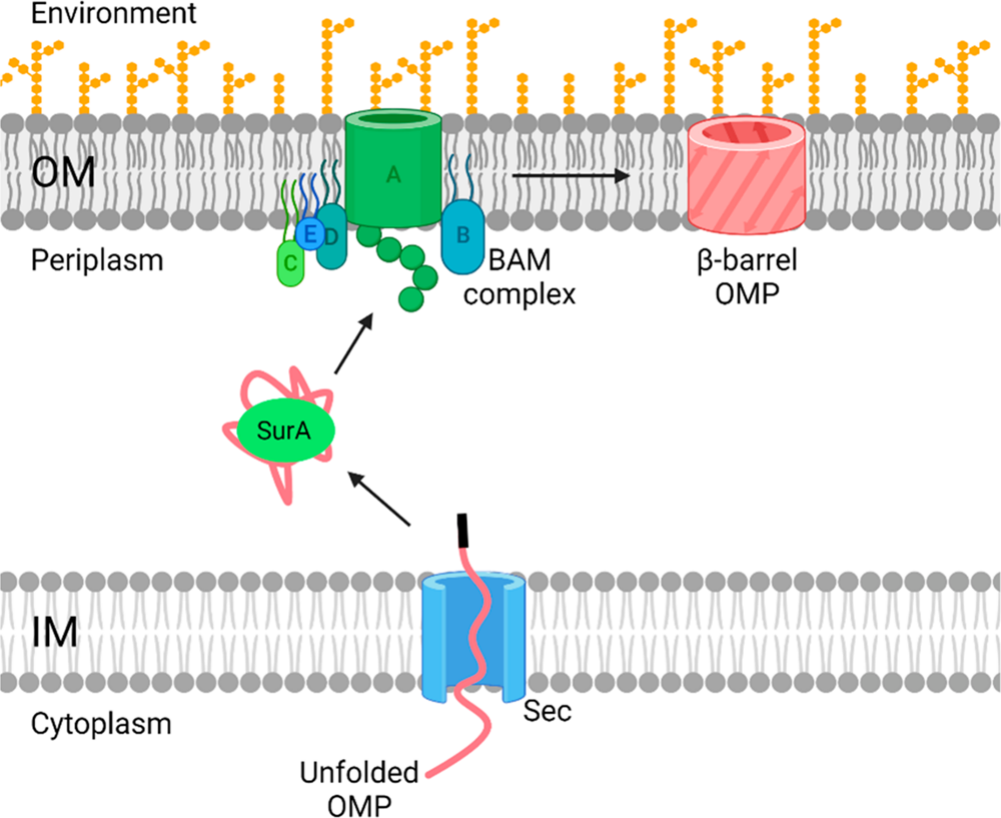
Schematic representation of β-barrel outer membrane protein (OMP) biogenesis. Unfolded OMPs are formed in the cytoplasm and are transported to the inner membrane (IM). The unfolded OMP moves into the periplasm through the Sec protein and is transported to the outer membrane (OM) via the chaperone protein, SurA. At the OM, the unfolded OMP enters the BAM complex which processes the protein. The BAM complex then releases the newly folded β-barrel protein into the OM.

A number of small-molecule BAM complex inhibitors have been reported in recent years (Figure 2).^14^ In 2019, researchers at Merck discovered the bis-guanidine MRL-494 (**1**) by screening for compounds that display antibacterial activity without crossing the OM.^15^ Mechanistic studies subsequently revealed that MRL-494 (**1**) kills Gram-negative bacteria by interfering with BAM-mediated OMP maturation. In the same year, Lewis and co-workers reported the first BamA-targeting natural product, darobactin (**2**).^16^ Darobactin binds with high affinity to the lateral gate of BamA, outcompeting the β-signal of unfolded OMPs, and in doing so blocks the first step of insertion of OMPs by BamA.^17^ As noted above, interference with OMP maturation can destabilize the bacterial cell envelope and in turn activate stress response pathways. Steenhuis et al. recently described the development of live-cell fluorescence-based screen assays that provide real-time reporting on the activation of the σ^e^ and the Rcs pathways, both of which are triggered in response to compounds that inhibit BAM complex activity.^18,19^ Application of these assays in high-throughput screening (HTS) campaigns led to the discoveries of VUF15259 (**3**) and compounds **4** and **5** as potential BAM inhibitors. In addition to such screening approaches, researchers at Polyphor recently disclosed a series of chimeric peptidomimetic antibiotics that target BAM, typified by compound **6**.^20^ These bicyclic peptide conjugates consist of a polymyxin E nonapeptide (PMEN) unit connected to a β-hairpin peptidomimetic derived from Polyphor’s previously developed murepavidin.^21^ While individually neither of the peptide monocycles exhibits significant antibacterial activity or interaction with the BAM complex, when they are covalently linked, the resulting chimeric species show potent bacterial killing that was subsequently revealed to be mediated by binding to BamA.^20−22^

**Figure 2:**
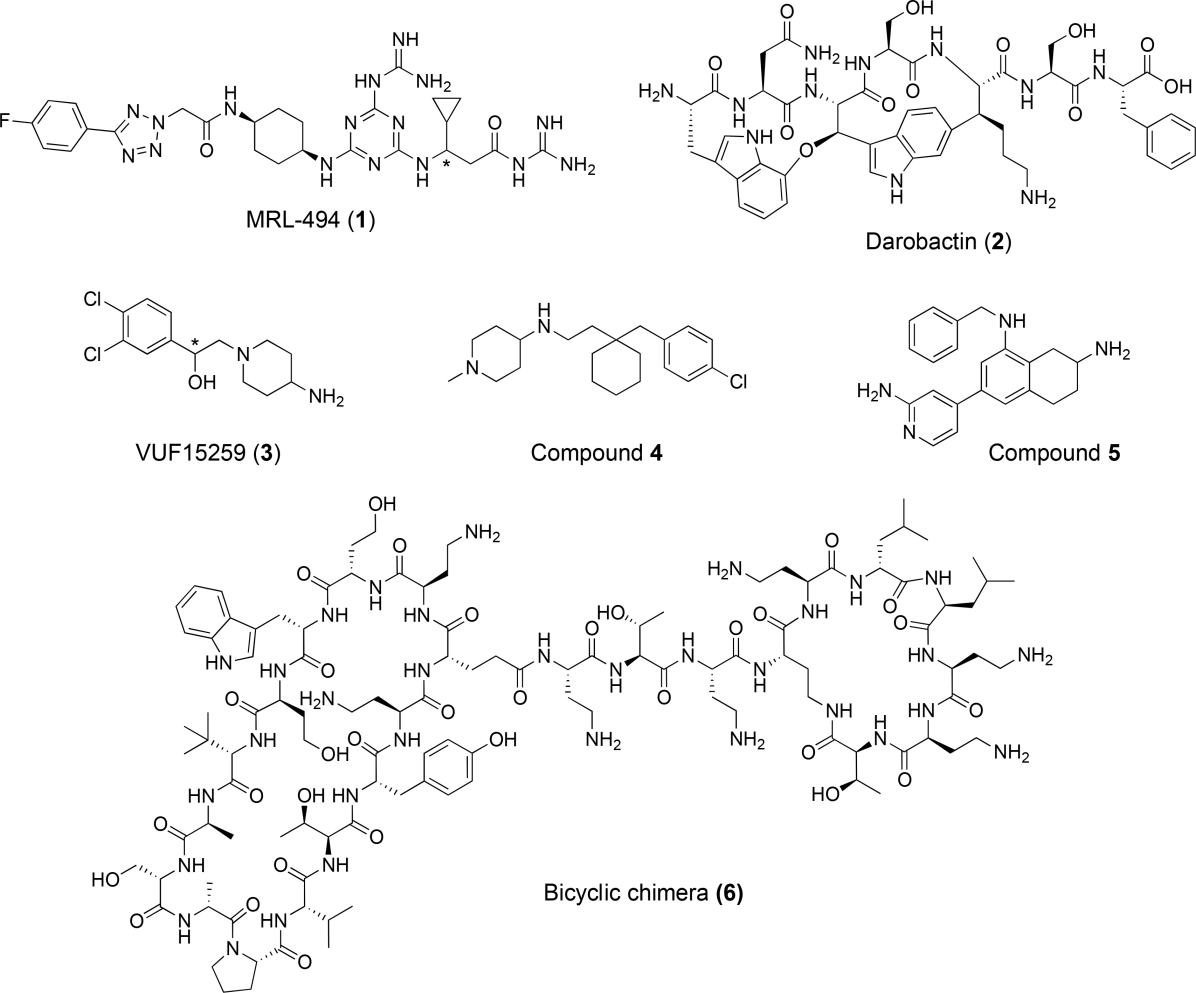
Reported BAM complex inhibitors: MRL-494 (**1**),^15^ darobactin (**2**),^16^ VUF15259 (**3**),^18^**4**, **5**,^19^ and **6**.^20^ MRL-494 (**1**) and VUF15259 (**3**) are both reported as racemic mixtures at the position denoted with *.

Interestingly, while MRL-494 (**1**) is the first reported BAM inhibitor, its discovery was rather serendipitous, given that the initial screen by which it was identified revealed the compound to in fact be an unintended byproduct.^15^ It is perhaps for this reason that, while a number of mechanistic studies have been performed with MRL-494 (**1**), no synthetic route for the preparation of the compound has yet been reported. In addition, while the current body of evidence strongly supports BAM as the target for MRL-494 (**1**), a precise molecular-level understanding of the structural requirements for this activity is lacking. Among the strongest lines of evidence that MRL-494 (**1**) interacts with BAM is the discovery of a resistant mutant containing a substitution in the BamA β-barrel, wherein a negatively charged glutamic acid at position 470 is mutated to a positively charged lysine.^15^ Interestingly, cellular thermal shift analyses of wild-type BamA and the E470K mutant concluded that both forms are thermally stabilized with MRL-494 (**1**) as a ligand. Recent investigations by Silhavy and co-workers have further shown that strains bearing the BamA^E470K^ mutation do not require BamD for OMP folding activity.^23^

Given the intriguing activity of MRL-494 (**1**) and the growing interest in BAM inhibitors in general, we were inspired to pursue a synthetic route for the preparation of MRL-494 (**1**) that could also be applied to generate analogues as a means of gaining structure–activity insights. Specifically, we were interested in examining the role played by the two guanidine moieties found in MRL-494 (**1**). To this end, structural variants lacking one or both of the guanidine groups were also prepared. The activities of the parent compound and the new analogues were assessed against a range of bacterial strains, focusing primarily on the Gram-negative members of the ESKAPE family. Synergy studies were also carried out by means of checkerboard assays to examine the potentiation of rifampicin against Gram-negative strains. In addition, the MRL-494 compounds were further assessed for their capacity to cause membrane disruption and induce bacterial stress response.

## Synthesis of MRL-494 (**1**) and Analogues

**Scheme 1:**
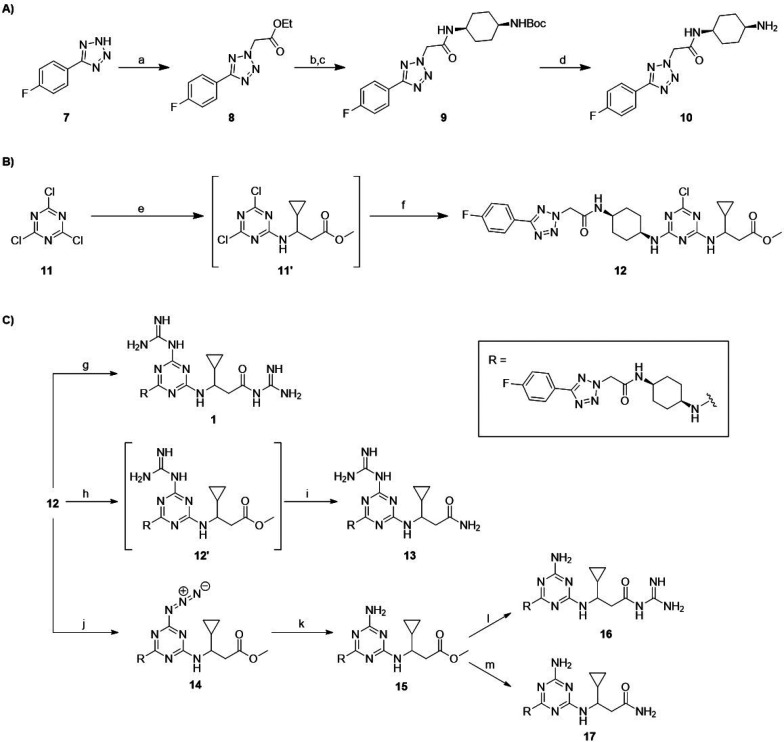
(A) Synthesis of Building Block **10**, (B) Synthesis of Scaffold **12**, and (C) Synthesis of MRL-494 (**1**) and Analogues **13**, **16**, and **17** Reagents and conditions for (A): (a) bromoethyl acetate, NaOEt, EtOH, 70 °C, 18 h; (b) 1 M NaOH, THF, rt, 18 h (72% over two steps); (c) 1-*N*-Boc-*cis*-1,4-cyclohexanediamine, NEt_3_, HBTU, DCM, rt, 18 h (90%); (d) TFA, DCM, rt, 3 h (quant). Reagents and conditions for (B): (e) (±)-methyl 3-amino-3-cyclopropylpropanoate·HCl, DIPEA, ACN, −10 °C to rt, 2 h; (f) **10**, DIPEA, ACN, rt, 18 h (55% over two steps). Reagents and conditions for (C): (g) guanidine·HCl, NaH, DABCO, DMF, rt, 18 h (54%); (h) guanidine·HCl, NaH, DABCO, DMF, rt, 18 h; (i) 7 M NH_3_ in MeOH, DABCO, 65 °C, 96 h (35% over two steps); (j) NaN_3_, DMF, 80 °C, 18 h (51%); (k) PPh_3_, pyridine, H_2_O, 55 °C, 18 h (50%); (l) guanidine·HCl, NaH, DABCO, DMF, rt, 72 h (51%); (m) 7 M NH_3_ in MeOH, DABCO, 65 °C, 2 wks (41%).

As illustrated in Scheme 1, the synthetic route developed for MRL-494 (**1**) and its analogues (compounds **13**, **16**, and **17**), prepared as racemic mixtures, comprises three stages: (A) the synthesis of building block **10**; (B) the assembly of common scaffold **12**; and (C) the addition of the amine or guanidine groups to produce the final products. To produce building block **10**, commercially available 5-(4′-fluorophenyl)-1*H*-tetrazole (**7**) was heated with bromoethyl acetate to yield **8**. The resulting ester was saponified with sodium hydroxide and subsequently coupled to 1-*N*-Boc-*cis*-1,4-cyclohexanediamine to yield **9**. The final step was the removal of the Boc protecting group under acidic conditions to give building block **10**. Common scaffold **12** was produced by controlled substitution of the chlorine groups on cyanuric chloride (**11**). The first substitution was carried out at −10 °C with (±)-methyl 3-amino-3-cyclopropylpropanoate·HCl (preparation described in the Supporting Information) and DIPEA for 1 h, and then the mixture was slowly warmed to room temperature. To the same reaction pot was added a solution of compound **10**, and the resulting mixture was stirred overnight to produce the target chlorotriazine **12**. The scaffold was split three ways to produce MRL-494 (**1**) and three analogues (**13**, **16**, and **17**) by substituting the two modifiable units (the triazine chlorine and the ester methoxy moiety) with either guanidine or ammonia. For each reaction involving the addition of a guanidine group, guanidine free base was used which was pre-prepared by mixing guanidine·HCl with an equimolar amount of sodium hydride. MRL-494 (**1**) was formed by mixing intermediate **12** with an excess of guanidine free base and a catalytic amount of DABCO to substitute both modifiable units. To produce analogue **13**, the guanidine group was selectively installed on the triazine portion of **12** by using equimolar amounts of guanidine free base. The solvent was removed, and the intermediate product was warmed to 65 °C in 7 M ammonia in MeOH, resulting in full conversion to **13**. Analogues **16** and **17** both contain an amino group on the triazine, which was installed by reacting **12** with sodium azide followed by the reduction of intermediate **14** to amine **15** using triphenylphosphine. Analogue **16** was then produced by reacting methyl ester with guanidine free base at 65 °C. By comparison, the conversion of intermediate **15** to analogue **17** was found to be very sluggish, with the desired product formed in reasonable yield after dissolving **15** in 7 M ammonia in MeOH and heating to 65 °C in a pressurized vessel for 2 weeks. Final purification of MRL-494 (**1**) and analogues **13**, **16**, and **17** was in all cases performed using RP-HPLC, providing the compounds in >95% purity.

## Antibacterial Activity Assays

We next assessed the antibacterial activity of MRL-494 (**1**) and analogues **13**, **16**, and **17** by determining their minimum inhibitory concentration (MIC) values against a panel of Gram-negative bacteria (Table 1). In agreement with published MIC data,^15^ MRL-494 (**1**) was found to exhibit antibacterial activity against four out of the five strains tested, with MIC values ranging from 8 to 32 μg/mL. Interestingly, this compound shows no activity against *K. pneumoniae* ATCC 13883 at the highest concentration tested. Analogues **13**, **16**, and **17** were not active against any of the strains tested, indicating that both guanidine groups are essential for antibacterial activity. The original report describing the discovery of MRL-494 (**1**) also noted that the compound possesses anti-Gram-positive activity.^15^ To this end the compounds were also tested against two Gram-positive strains, *MSSA* 29213 and *MRSA* USA 300 (see Supporting Information Table S1). In line with our expectation, MRL-494 (**1**) was found to have an MIC of 8 μg/mL against both strains, while analogues **13** and **16** were both found to exhibit MIC values of 64 and 128 μg/mL against these strain, respectively. Analogue **17**, in which both guanidine groups are replaced by the corresponding amino moiety, showed no antibacterial activity against either Gram-positive strain.

**Table 1:** Antibacterial Activity of MRL-494 (**1**) and Analogues **13**, **16**, and **17** against Various Gram-Negative Strains

	MIC^a^			
strain	MRL-494 (**1**)	**13**	**16**	**17**
*E. coli* ATCC 25922	16	>128	128	>128
*E. coli* BW25113^b^	8	128	128	>128
*K. pneumoniae* ATCC 13883	>128	>128	>128	>128
*A. baumannii* ATCC 9955	32	>128	>128	>128
*P. aeruginosa* ATCC 27853	16	128	128	>128

a Minimum inhibitory concentration (μg/mL). Results are an average of three technical replicates.

b Standard lab strain.

**Figure 3:**
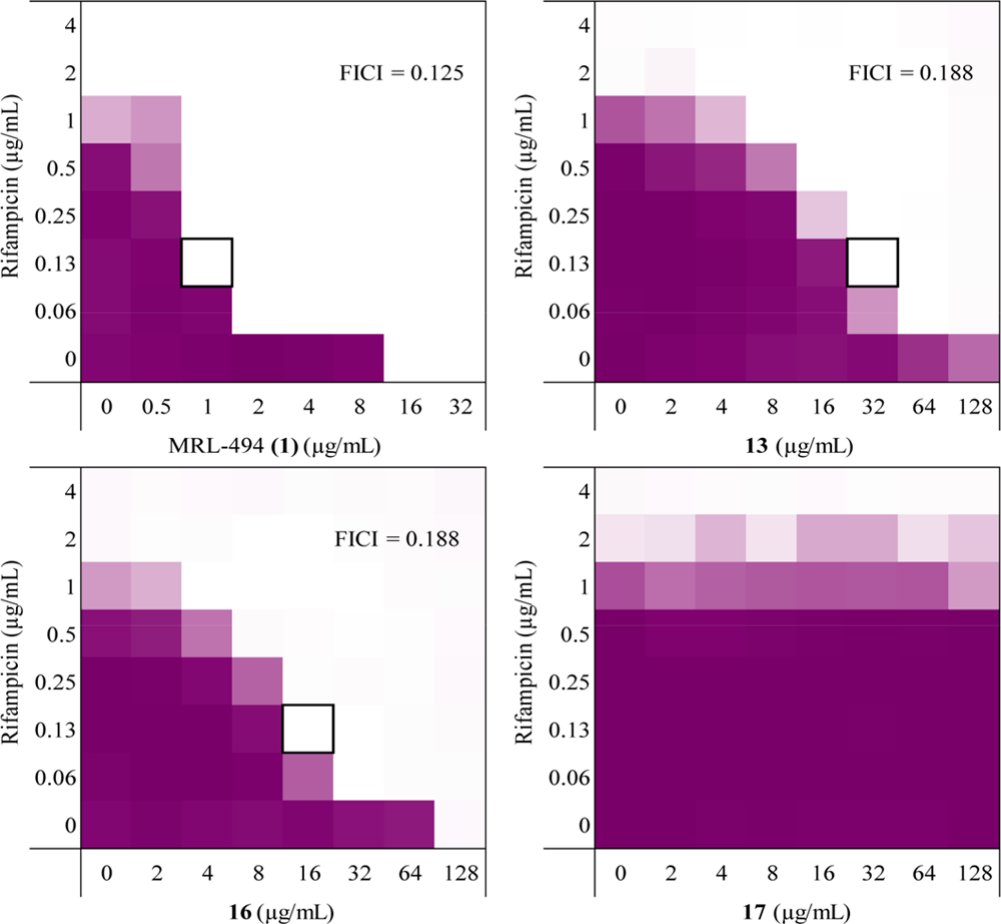
Checkerboard assay results for MRL-494 (**1**) and analogues **13**, **16**, and **17** in combination with rifampicin against *E. coli* ATCC 25922 (see Supporting Information Figures S1–S4 for checkerboard assays with other strains). The combination of test compound and rifampicin which resulted in the lowest FICI is indicated by a black box. The mean optical density of the bacterial growth (OD_600_) is shown as a color gradient, with purple signifying maximum bacterial growth and white as no growth.

MRL-494 (**1**) was also reported to show synergistic activity against Gram-negative bacteria when paired with rifampicin, an antibiotic that is typically only active against Gram-positive strains.^15^ To investigate this synergistic effect further, we carried out a series of checkerboard assays wherein MRL-494 (**1**) or analogues **13**, **16**, and **17** were evaluated in combination with rifampicin against a panel of Gram-negative strains (Figure 3 and Supporting Information Figures S1–S4). Checkerboard assays allow for the calculation of the fractional inhibitory concentration index (FICI) of a given combination, and in cases where a combination exhibits an FICI value of ≤0.5, it is said to be synergistic.

MRL-494 (**1**) was found to synergize well with rifampicin against each of the strains tested, with FICI values of <0.3 in all cases (Table 2). Of note is the FICI value determined against *K. pneumoniae* ATCC 13883. Despite MRL-494 (**1**) having no intrinsic antibacterial activity against this strain, it is able to synergize very well with rifampicin, with an FICI value of ≤0.039. The synergistic activity of the MRL-494 analogues prepared was also assessed (Supporting Information Tables S2–S4). This showed the analogues containing at least one guanidine group (compounds **13** and **16**) to be effective synergists, with both resulting in FICI values <0.3 for four out of five strains, the only exception being *P. aeruginosa* ATCC 27853. Against this strain, neither compound was able to synergize with rifampicin. In contrast, analogue **17**, lacking both guanidine moieties, showed no capacity to synergize with rifampicin against any of the strains tested. Taken together, this data indicates that at least one of the guanidine groups needs to be present for synergistic activity. Also, while the FICI values measured for MRL-494 (**1**) and analogues **13** and **16** are similar, a much lower concentration of MRL-494 (**1**) results in an FICI <0.5, making it the more potent synergist.

**Table 2:** Results of Checkerboard Assays with MRL-494 (**1**) and Rifampicin

	MIC^a^				
	MRL-494 (**1**)		rifampicin		
strain	alone	in combination	alone	in combination	FICI^b^
*E. coli* ATCC 25922	16	1	2	0.13	0.125
*E. coli* BW25113	8	2	4	0.13	0.281
*K. pneumoniae* ATCC 13883	>128	2	8	0.25	≤0.039
*A. baumannii* ATCC 9955	32	2	1	0.06	0.125
*P. aeruginosa* ATCC 27853	16	4	16	0.25	0.266

a Minimum inhibitory concentration (μg/mL).

b Synergy defined as FICI ≤0.5.

## Outer Membrane Permeabilization Assay

**Figure 4:**
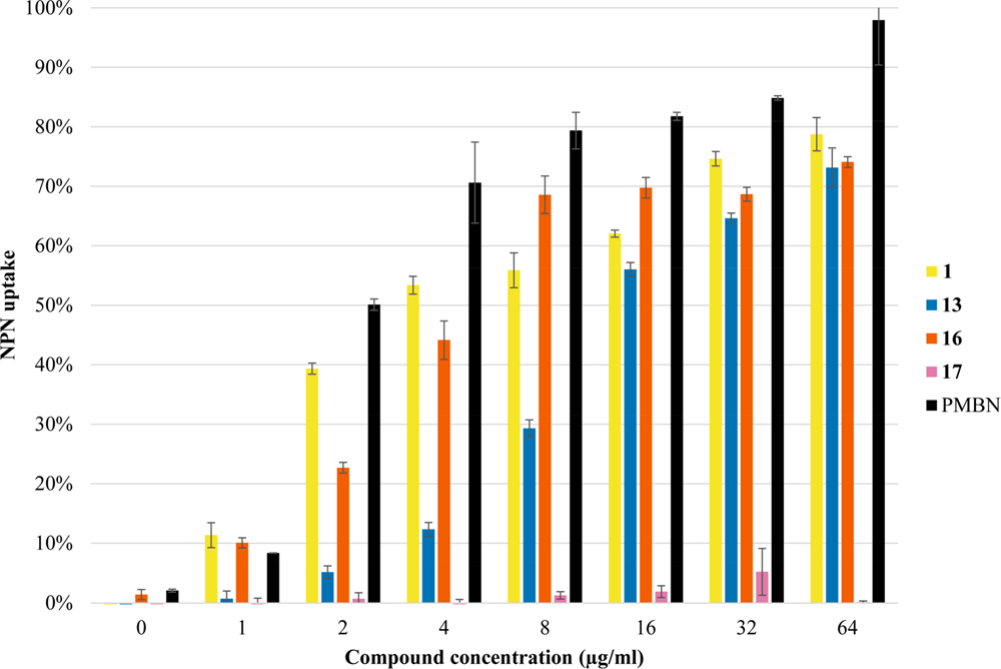
Results from the fluorescence-based OM permeabilization assay of MRL-494 (**1**) and analogues **13**, **16**, and **17** against *E. coli* BW25113. Fluorescence of *N*-phenylnaphthalen-1-amine (NPN) was read using a plate reader with λ_ex_ = 355 nm and λ_em_ = 420 nm after 60 min of incubation. The NPN uptake values shown are calculated relative to the uptake obtained when the cells are treated with colistin (100 μg/mL). The values are also corrected for the background signal determined by the negative control (DMSO). Error bars represent the standard deviation based on technical replicates (*n* = 3).

The ability of MRL-494 (**1**) to potentiate the activity of rifampicin suggests that it may be able to permeabilize the OM. To study this in more detail, we used an established fluorescence-based assay to assess the capacity for MRL-494 (**1**) and analogues **13**, **16**, and **17** to cause OM permeabilization.^24,25^ This assay makes use of *N*-phenylnaphthalen-1-amine (NPN), a compound that changes fluorescence depending on the polarity of its surrounding environment. In the presence of intact Gram-negative bacterial cells in an aqueous environment, NPN is weakly fluorescent, but if the OM is disturbed, the NPN can penetrate into the nonpolar phospholipid bilayer, resulting in a measurable increase in fluorescence. In this experiment DMSO was employed as negative control and the known OM permeabilizing antibiotic colistin was used as a positive control. Polymyxin B nonapeptide (PMBN) was also tested alongside our compounds as a representative compound with no antibacterial activity but the ability to disrupt the OM. In line with the results of the rifampicin synergy studies, MRL-494 (**1**) and analogues **13** and **16** were found to effectively permeabilize the OM, as indicated by their ability to induce NPN uptake (Figure 4). The three compounds exhibit a dose-dependent increase in fluorescence, indicating an increase in OM permeabilization at higher concentrations. Notably, compound **13** does not permeabilize the membrane well at lower concentrations when compared with MRL-494 (**1**) or **16**, indicating that the positioning of the guanidine group influences the compound’s ability to interact with the OM. Conversely, and also in agreement with the results of the activity and synergy assays, analogue **17** was found to cause very little OM permeabilization. The membranolytic effects of positively charged moieties are also well recognized, and so the presence of guanidine groups, or lack thereof, in MRL-494 and the analogues here studied may also provide an explanation for these findings.^26−28^ To assess the specificity of the OM disruption caused by MRL-494 (**1**) and analogues **13** and **16**, we also tested their hemolytic activity (Supporting Information Figure S5 and Table S5). Only at the highest concentrations tested was MRL-494 (**1**) found to be weakly hemolytic (6.8% at 64 μg/mL and 23.4% at 128 μg/mL), while analogues **13** and **16** did not display hemolytic behavior.

## Evaluating Rcs Stress Response

**Figure 5:**
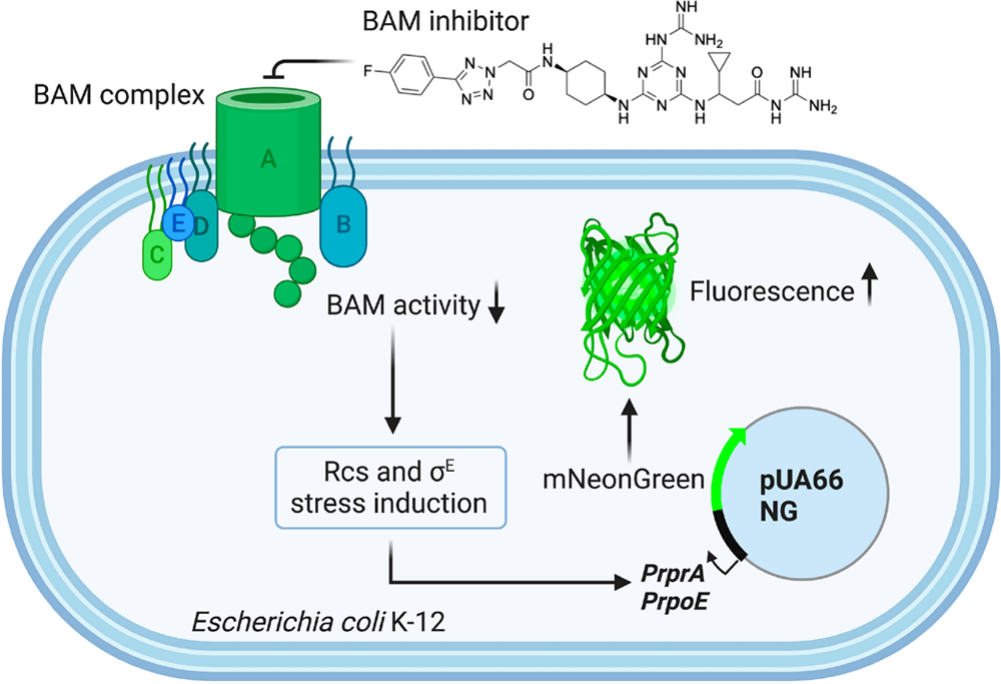
Rcs stress response assay employing fluorescent *E. coli* K-12 strain engineered to report on activation of Rcs stress response induced upon exposure to BAM inhibitors.

We next assessed the ability of MRL-494 (**1**) and its analogues to induce bacterial stress responses associated with impaired BAM activity. The Rcs (Regulation of capsular polysaccharide synthesis) response is particularly sensitive toward impaired functioning of the BAM complex and also responds to perturbations in the biogenesis of peptidoglycan, lipoproteins, and lipopolysaccharides.^29^ Although the underlying molecular mechanisms are not yet fully elucidated, many inducing cues are signaled through the sensor protein RcsF, which is a surface-exposed OM lipoprotein. To identify novel agents that affect diverse aspects of OM biogenesis and integrity, we recently developed whole-cell fluorescence-based HTS assays that report on Rcs, Cpx, and σ^E^ cell envelope stress (Figure 5).^30,31^ Using these assays, we have demonstrated that perturbations of specific OM processes produce unique stress reporter profiles that can be exploited for drug screening purposes and can specifically detect compounds that inhibit BamA.^18,19^ To this end we used our Rcs stress response assay to evaluate whether MRL-494 (**1**) and analogues **13**, **16**, and **17** are able to induce the Rcs stress response.

**Figure 6:**
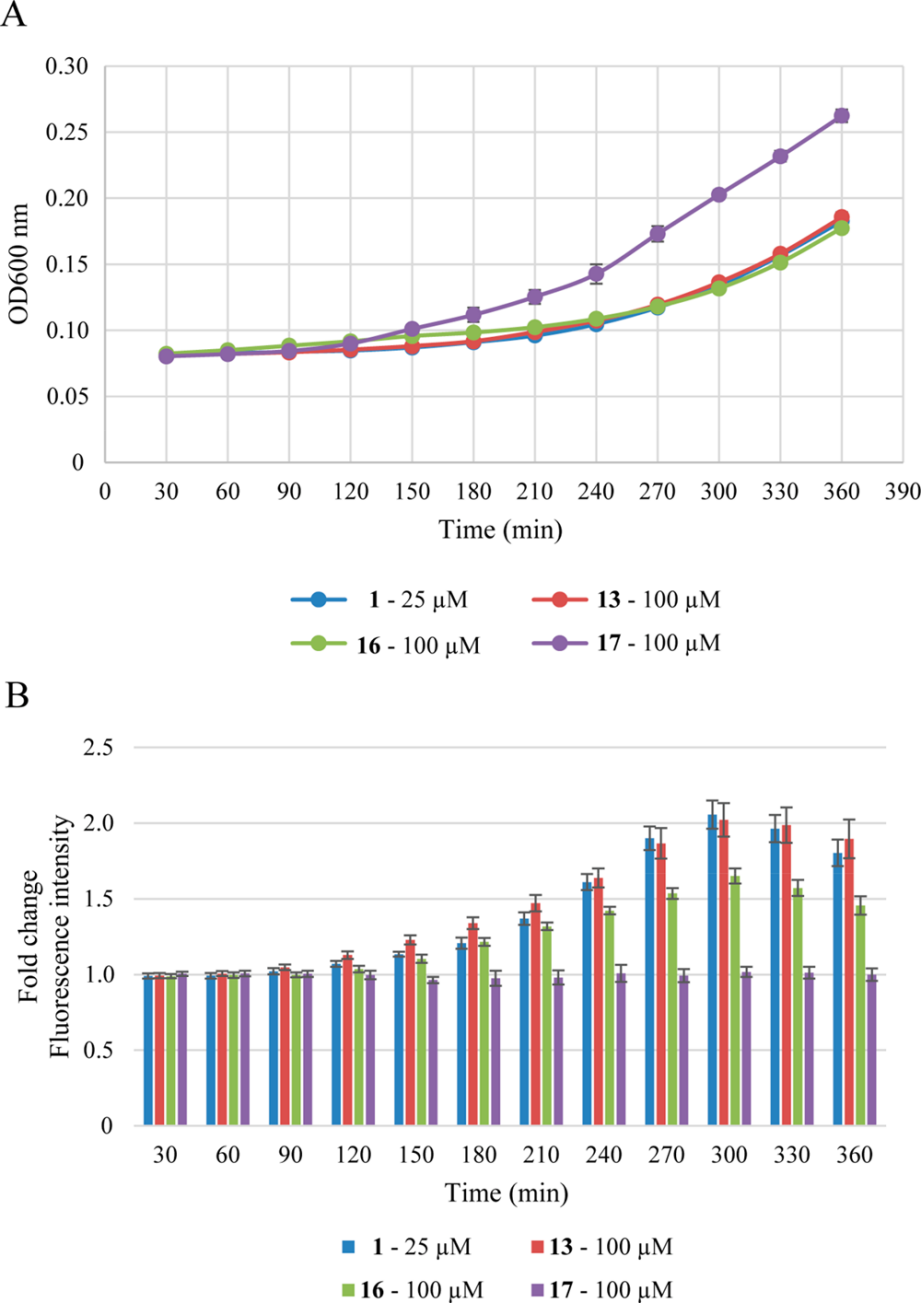
Real-time monitoring of bacterial growth and Rcs stress activation in response to MRL-494 (**1**) and analogues **13**, **16**, and **17**. *E. coli* TOP10F′ cells, harboring the PrprA-mNG reporter construct, were grown in a 96-well plate and exposed to the compounds at the indicated concentration at time point 0. Growth (A; OD_600_) and mNG fluorescence (B) were measured in time. Fluorescence was corrected for growth (OD_600_) and plotted as fold-change of signal compared to untreated cells (set to 1). Error bars represent the standard deviation of triplicate technical replicates.

In doing so, *E. coli* Top10F′ cells harboring the Rcs response reporter plasmid were grown in 96-well plates containing a 2-fold increasing concentration of the compounds up to 200 μM. The effect of the compounds on fluorescence and growth (optical density at 600 nm) was followed in real time. With respect to growth, the reporter strain appeared most sensitive to MRL-494 (**1**) and insensitive to compound **17**, even at the highest concentration tested (Supporting Information Figures S6 and S7), consistent with the effect on other *E. coli* strains analyzed (Table 1). At the highest sublethal concentration tested (25 μM), MRL-494 (**1**) mounted a significant (∼2 fold) Rcs signal, as expected (Figure 6B), even exceeding the signal elicited by the positive control compound VUF15259 (**3**)^18^ (Supporting Information Figure S7). At the same 25 μM concentration the Rcs signal was very limited for compounds **13** and **16** and undetectable for compound **17** (Supporting Information Figures S6 and S7). At a concentration of 100 μM, however, compounds **13** and **16** provoked a similar growth defect as MRL-494 (**1**) at 25 μM (Figure 6A). Importantly, this was accompanied by a significant Rcs signal following similar kinetics, although slightly less in amplitude for compound **16** (Figure 6B). In contrast, no Rcs signal was detected for compound **17** at any concentration tested (Supporting Information Figure S7). Together, the data are consistent with a gradual loss in activity of compounds **13** and **16** that yet likely act on the same target as MRL-494 (**1**), while compound **17** has lost all activity.

In summary, we here describe the synthesis of the BAM complex inhibitor MRL-494 (**1**) via a route that is both robust and scalable, providing ready access to the compound in multi-hundred milligram quantities. Given its modular nature, the route also provides ready access to analogues, which allowed us to probe the necessity of the two guanidine groups present in MRL-494. The rationale for exploring the role of these guanidine moieties was inspired by reports that resistance to MRL-494 (**1**) is conferred by a mutation in BamA of Glu470 to Lys. Given that guanidine groups can effectively hydrogen bond with carboxylates, we hypothesized that an interaction of the Glu470 side chain with either guanidino group of MRL-494 (**1**) might be key for its activity, leading us to generate analogues **13**, **16**, and **17**. The activity of MRL-494 (**1**) and these analogues was in turn assessed against a panel of Gram-negative bacteria, revealing that both guanidine groups are necessary for antibacterial activity. We also investigated the synergistic capabilities of MRL-494 (**1**) with rifampicin by way of checkerboard assays which revealed MRL-494 (**1**) to be a potent synergist. Interestingly, we discovered that synergistic activity is retained in the analogues bearing a single guanidine group. We also found that MRL-494 (**1**) and analogues **13** and **16** cause OM permeabilization at concentrations much lower than those that induce hemolytic activity. Finally, we also provide new evidence in support of a BAM-targeted mechanism of action for MRL-494 (**1**) by demonstrating its capacity to induce a cellular stress response in a recently developed assay used to identify compounds that inhibit BAM.

## Methods

### General Procedures

All reagents used were of American Chemical Society (ACS) grade or finer and were used as received without any further purification. ^1^H and ^13^C NMR spectra were recorded on a Bruker AV-400 MHz or AV-500 MHz instrument. Checkerboards, NPN assay, and hemolysis were analyzed by a Tecan Spark plate reader. High-resolution mass spectrometry (HRMS) analyses were performed on a Shimadzu Nexera X2 UHPLC system. For full description of analytical methods, see the Supporting Information.

### Synthesis

#### Ethyl 2-(5-(4-Fluorophenyl)-2*H*-tetrazol-2-yl)acetate (**8**)

5-(4′-Fluorophenyl)-1*H*-tetrazole (2.00 g, 12.2 mmol, 1 equiv) was dissolved in EtOH (50 mL) along with NaOEt (870 mg, 12.8 mmol, 1.05 equiv). Bromoethyl acetate (1.35 mL, 12.2 mmol, 1 equiv) was added dropwise to the solution, and the reaction mixture was refluxed overnight at 90 °C. After 18 h the solution was filtered while still hot, and the filtrate was concentrated. No further purification was done, and the solid was used directly in the next reaction (5.25 g, quant.). Synthesized as previously described, and data gathered was consistent with that published.^32^

^1^H NMR (400 MHz, CDCl_3_) δ 8.17–8.12 (m, 2H), 7.20–7.14 (m, 2H), 5.44 (s, 2H), 4.29 (q, *J* = 7.1 Hz, 2H), 1.29 (t, *J* = 7.1 Hz, 3H). ^13^C NMR (101 MHz, CDCl_3_) δ 165.1, 164.8, 164.2 (d, *J* = 250.5 Hz), 129.0 (d, *J* = 8.7 Hz), 123.3 (d, *J* = 3.3 Hz), 116.1 (d, *J* = 22.0 Hz), 62.8, 53.4, 14.1. HRMS (ESI): calculated for C_11_H_12_FN_4_O_2_ [M+H]^+^ 251.0939, found 251.0941.

#### *tert*-Butyl ((1s,4s)-4-(2-(5-(4-Fluorophenyl)-2*H*-tetrazol-2-yl)acetamido)cyclohexyl)carbamate (**9**)

Compound **8** (5.25 g, 12.2 mmol, 1 equiv) was dissolved in THF (30 mL) before NaOH (18 mL, 1 M) was added and stirred overnight. The reaction mixture was partitioned between water (30 mL) and EtOAc (30 mL) before acidifying the water layer to pH 3 using 5 N HCl. The product was extracted from the water layer with EtOAc (3 × 40 mL), and the organic layer was dried using sodium sulfate and concentrated. The resulting solid (2.7 g, quant.) was used directly in the next reaction. The intermediate acid (1.04 g, 4.67 mmol, 1 equiv), 1-*N*-Boc-*cis*-1,4-cyclohexanediamine (1 g, 4.67 mmol, 1 equiv), and NEt_3_ (1.95 mL, 14.01 mmol, 3 equiv) were dissolved in DCM (40 mL). HBTU (3.54 g, 9.34 mmol, 2 equiv) was added and stirred for 18 h. When the reaction was complete by TLC (1:1 PE/EtOAc), the reaction mixture was partitioned between water (40 mL) and DCM and the aqueous layer extracted with DCM (2 × 150 mL). The combined organic layers were washed with brine, dried over sodium sulfate, and concentrated. The resulting solid was silica column purified (1.5:1 to 1:1.25, PE/EtOAc) to obtain the desired product (1.75 g, 90%).

^1^H NMR (400 MHz, CDCl_3_) δ 8.21–8.12 (m, 2H), 7.26–7.15 (m, 2H), 6.35 (s, 1H), 5.38 (s, 2H), 4.57 (s, 1H), 3.95 (tt, *J* = 7.1, 4.0 Hz, 1H), 3.60 (s, 1H), 1.73 (tt, *J* = 11.1, 8.6, 4.1 Hz, 4H), 1.55 (m, 4H), 1.44 (s, 9H). ^13^C NMR (101 MHz, CDCl_3_) δ 165.1, 164.6 (d, *J* = 250.9 Hz), 162.8, 129.1 (d, *J* = 8.7 Hz), 123.0 (d, *J* = 3.1 Hz), 116.4 (d, *J* = 22.1 Hz), 77.5, 77.2, 76.8, 55.6, 46.9, 28.6, 28.5, 28.0. HRMS (ESI): calculated for C_11_H_12_FN_4_O_2_ [M+H]^+^ 419.2202, found 419.2203.

#### *N*-((1s,4s)-4-Aminocyclohexyl)-2-(5-(4-fluorophenyl)-2*H*-tetrazol-2-yl)acetamide (**10**)

Intermediate **9** (1.74 g, 4.18 mmol, 1 equiv) was dissolved in DCM (20 mL). TFA (10 mL) was added to the solution along with a few drops of water. The reaction was monitored by TLC and was deemed complete with the consumption of the starting material (1:1 PE/EtOAc). The solvent was removed and the resulting oil was used directly in the next reaction without further purification (1.508 g, quant., yield was assumed to be quantitative and weight of salt was considered in the next step).

^1^H NMR (400 MHz, MeOD) δ 8.18–8.12 (m, 1H), 7.31–7.23 (m, 2H), 5.52 (s, 2H), 3.99–3.92 (m, 1H), 3.27–3.20 (m, 1H), 1.98–1.84 (m, 4H), 1.84–1.70 (m, 2H). ^13^C NMR (101 MHz, MeOD) δ 166.4, 165.8 (d, *J* = 250.2 Hz), 165.6, 130.0 (d, *J* = 8.7 Hz), 125.0 (d, *J* = 3.3 Hz), 117.1 (d, *J* = 22.3 Hz), 55.7, 49.9, 46.6, 28.1, 26.9. HRMS (ESI): calculated for C_15_H_20_FN_6_O [M+H]^+^ 319.1677, found 319.1679.

#### Methyl 3-((4-Chloro-6-(((1s,4s)-4-(2-(5-(4-fluorophenyl)-2*H*-tetrazol-2-yl)acetamido)cyclohexyl)amino)-1,3,5-triazin-2-yl)amino)-3-cyclopropylpropanoate (**12**)

Cyanuric chloride (114 mg, 1.12 mmol, 1 equiv) was dissolved in acetonitrile (7 mL) and cooled with an ice/brine bath. (±)-Methyl 3-amino-3-cyclopropylpropanoate·HCl (preparation described in the Supporting Information) (200 mg, 1.12 mmol, 1 equiv) was added followed by DIPEA (800 μL, 4.48 mmol, 4 equiv). The reaction was stirred for 1 h at −10 °C followed by an hour at room temperature. Intermediate **10** (432 mg, 1.12 mmol, 1 equiv) dissolved in acetonitrile (3 mL) and DIPEA (800 μL, 4.48 mmol, 4 equiv) were added dropwise to the solution and stirred overnight. The reaction was monitored by TLC (49:1 DCM/MeOH). Once complete, the reaction mixture was washed with 1 N HCl (3 × 5 mL) and then brine (3 × 5 mL). The desired product (339 mg, 52%) was obtained by silica column chromatography (49:1 to 19:1 DCM/MeOH). ^1^H NMR (400 MHz, MeOD) δ 8.18–8.12 (m, 2H), 7.30–7.23 (m, 2H), 5.50 (s, 2H), 3.92 (d, *J* = 13.1 Hz, 2H), 3.77 (dt, *J* = 8.7, 6.3 Hz, 1H), 3.70–3.59 (m, 3H), 2.83–2.56 (m, 2H), 1.77 (d, *J* = 9.4, 3.8 Hz, 8H), 1.13–0.99 (m, 1H), 0.59–0.45 (m, 2H), 0.43–0.34 (m, 1H), 0.32–0.21 (m, 1H). ^13^C NMR (101 MHz, MeOD) δ 173.5, 166.0, 165.7, 165.6 (d, *J* = 249.0 Hz), 130.1 (d, *J* = 8.6 Hz), 125.0 (d, *J* = 3.4 Hz), 117.1 (d, *J* = 22.4 Hz), 55.8, 54.2, 53.5, 52.2, 48.0, 40.8, 40.4, 28.9, 28.8, 16.8, 16.5, 4.2, 4.1, 3.8, 3.5. HRMS (ESI): calculated for C_25_H_31_ClFN_10_O_3_ [M+H]^+^ 573.2248, found 573.2251.

#### *N*-Carbamimidoyl-3-cyclopropyl-3-((4-(((1s,4s)-4-(2-(5-(4-fluorophenyl)-2*H*-tetrazol-2-yl)acetamido)cyclohexyl)amino)-6-guanidino-1,3,5-triazin-2-yl)amino)propanamide, MRL-494 (**1**)

A guanidine solution was prepared by mixing guanidine hydrochloride (100 mg, 1.05 mmol) and NaH (60% w/w oil dispersion, 42 mg, 1.05 mmol) in dry DMF (1 mL). Intermediate **12** (90 mg, 154 μmol, 1 equiv) and DABCO (17 mg, 172 μmol, 1 equiv) were dissolved in the guanidine free base solution (620 μL, 616 μmol, 4 equiv). The reaction mixture was stirred overnight and monitored by LCMS. When the starting material showed full conversion to the desired product, the reaction mixture was crashed out in water (10 mL) and the resulting solid washed with diethyl ether (3 × 10 mL). The crude material was HPLC prep purified (0–100% Buffer B over 60 min) and lyophilized to give a white powder (52 mg, 54%). Solvent system: Buffer A, 95:5:0.1 H_2_O/ACN/TFA; Buffer B, 95:5:0.1 ACN/H_2_O/TFA.

^1^H NMR (500 MHz, MeOD) δ 8.17–8.13 (m, 2H), 7.29–7.24 (m, 2H), 5.51 (d, *J* = 4.0 Hz, 2H), 3.95 (s, 1H), 3.92–3.85 (m, 2H), 2.88–2.83 (m, 2H), 1.78 (s, 8H), 1.14–1.07 (m, 1H), 0.60–0.52 (m, 2H), 0.39 (d, *J* = 4.8 Hz, 2H). ^13^C NMR (126 MHz, MeOD) δ 174.9, 166.2, 166.1, 165.7, 165.6 (d, *J* = 249.1 Hz), 164.0, 162.8, 157.6, 156.8, 130.1 (d, *J* = 8.7 Hz), 125.0 (d, *J* = 3.3 Hz), 117.1 (d, *J* = 22.4 Hz), 55.8, 53.6, 53.1, 48.2, 44.0, 43.9, 43.8, 29.0, 28.8, 16.9, 16.8, 4.2, 4.1, 3.9. HRMS (ESI): calculated for C_26_H_36_FN_16_O_2_ [M+H]^+^ 623.3186, found 623.3190.

#### 3-Cyclopropyl-3-((4-(((1s,4s)-4-(2-(5-(4-fluorophenyl)-2*H*-tetrazol-2-yl)acetamido)cyclohexyl)amino)-6-guanidino-1,3,5-triazin-2-yl)amino)propanamide (**13**)

Guanidine free base solution was prepared by mixing guanidine·HCl (100 mg, 1.05 mmol) and NaH (60% w/w oil dispersion, 42 mg, 1.05 mmol) in dry DMF (500 μL). Intermediate **12** (82 mg, 0.139 mmol, 1 equiv) and DABCO (15 mg, 0.139, 1 equiv) were dissolved in dry DMF (150 μL) before the addition of guanidine free base solution (67 μL, 0.139 mmol, 1 equiv). The reaction mixture was stirred overnight and monitored by LCMS. The solvent was removed by reduced pressure, and the resulting oil was redissolved in a vial with 7 M ammonia in MeOH (2 mL). The reaction was warmed to 65 °C and stirred for 72 h until the reaction was complete by LCMS. The organic solvent was removed, and the resulting solid was HPLC prep purified (0–100% Buffer B over 60 min) and then lyophilized to give a white powder (27 mg, 35%). Solvent system: Buffer A, 95:5:0.1 H_2_O/ACN/TFA; Buffer B, 95:5:0.1 ACN/H_2_O/TFA.

^1^H NMR (500 MHz, MeOD) δ 8.15 (m, 2H), 7.27 (m, 2H), 5.50 (s, 2H), 3.97 (d, *J* = 26.6 Hz, 1H), 3.88 (d, *J* = 7.3 Hz, 1H), 3.82–3.75 (m, 1H), 2.64–2.50 (m, 2H), 1.78 (s, 8H), 1.08–1.00 (m, 1H), 0.60–0.52 (m, 1H), 0.51–0.45 (m, 1H), 0.43–0.38 (m, 1H), 0.36–0.30 (m, 1H). ^13^C NMR (126 MHz, MeOD) δ 175.0, 164.7, 164.3, 164.2 (d, *J* = 248.9 Hz), 156.0, 128.7 (d, *J* = 8.6 Hz), 123.7, 115.8 (d, *J* = 22.2 Hz), 54.5, 52.4, 46.7, 40.8, 40.6, 27.8, 27.7, 27.5, 15.7, 2.8, 2.3. HRMS (ESI): calculated for C_25_H_34_FN_14_O_2_ [M+H]^+^ 581.2968, found 581.2970.

#### Methyl 3-((4-Azido-6-(((1s,4s)-4-(2-(5-(4-fluorophenyl)-2*H*-tetrazol-2-yl)acetamido)cyclohexyl)amino)-1,3,5-triazin-2-yl)amino)-3-cyclopropylpropanoate (**14**)

Intermediate **12** (484 mg, 0.8466 mmol, 1 equiv) was dissolved in DMF (2.5 mL) before sodium azide (66 mg, 1.015 mmol, 1.2 equiv) was added and the resulting solution warmed to 90 °C overnight. A further portion of sodium azide (66 mg, 1.015 mmol, 1.2 equiv) was added. TLC (19:1 DCM/MeOH) showed consumption of the starting material, and the solvent was removed. The crude material was silica column purified (49:1 to 24:1 DCM/MeOH) to give the desired product (250 mg, 51%).

^1^H NMR (400 MHz, CDCl_3_) δ 8.19–8.08 (m, 2H), 7.19 (t, *J* = 8.7 Hz, 2H), 6.43 (d, *J* = 7.6 Hz, 1H), 5.76 (d, *J* = 8.2 Hz, 0H), 5.38 (s, 2H), 5.25 (s, 0H), 3.97 (s, 2H), 3.72–3.59 (m, 4H), 2.84–2.58 (m, 2H), 1.83–1.69 (m, 4H), 1.69–1.47 (m, 4H), 1.06 (s, 1H), 0.57–0.44 (m, 2H), 0.44–0.31 (m, 1H), 0.31–0.20 (m, 1H). ^13^C NMR (101 MHz, CDCl_3_) δ 165.1, 164.3 (d, *J* = 251.1 Hz), 162.9, 129.1 (d, *J* = 8.7 Hz), 123.0 (d, *J* = 3.5 Hz), 116.3 (d, *J* = 22.1 Hz), 55.5, 51.8, 28.3, 27.8, 15.6, 15.5, 3.8, 3.8. HRMS (ESI): calculated for C_25_H_31_FN_13_O_3_ [M+H]^+^ 580.2652, found 580.2655.

#### Methyl 3-((4-Amino-6-(((1s,4s)-4-(2-(5-(4-fluorophenyl)-2*H*-tetrazol-2-yl)acetamido)cyclohexyl)amino)-1,3,5-triazin-2-yl)amino)-3-cyclopropylpropanoate (**15**)

Intermediate **14** (250 mg, 432 μmol, 1 equiv) was dissolved in a mix of pyridine/H_2_O (4.7 mL, 10:1). Triphenylphosphine (226 mg, 863 μmol, 2 equiv) was added and the reaction stirred for 48 h at 85 °C. LCMS showed complete consumption of starting material. The solvent was removed and the residue was redissolved in EtOAc (50 mL). The organic layer was washed with water (2 × 30 mL), dried with magnesium sulfate, and concentrated. The crude material was silica column purified (97:3 to 19:1 DCM/MeOH) to give the desired product (79 mg, 33%).

^1^H NMR (400 MHz, CDCl_3_) δ 8.14–8.07 (m, 2H), 7.15 (t, *J* = 8.6 Hz, 2H), 6.94 (s, 1H), 6.27 (s, 1H), 5.87 (s, 1H), 5.45 (s, 2H), 5.17 (s, 1H), 4.76 (s, 1H), 3.94 (s, 2H), 3.64 (s, 4H), 2.79–2.60 (m, 2H), 1.76–1.62 (m, 4H), 1.63–1.48 (m, 4H), 1.09–0.97 (m, 1H), 0.55–0.46 (m, 1H), 0.45–0.39 (m, 1H), 0.33 (s, 1H), 0.29–0.15 (m, 1H). ^13^C NMR (101 MHz, CDCl_3_) δ 172.4, 164.9, 164.3 (d, *J* = 250.8 Hz), 163.3, 129.1 (d, *J* = 8.6 Hz), 123.2, 116.3 (d, *J* = 22.0 Hz), 77.4, 55.5, 51.8, 46.8, 46.1, 39.8, 28.4, 27.8, 15.7, 3.9, 3.6. HRMS (ESI): calculated for C_25_H_33_FN_11_O_3_ [M+H]^+^ 554.2746, found 554.2750.

#### 3-((4-Amino-6-(((1s,4s)-4-(2-(5-(4-fluorophenyl)-2*H*-tetrazol-2-yl)acetamido)cyclohexyl)amino)-1,3,5-triazin-2-yl)amino)-*N*-carbamimidoyl-3-cyclopropylpropanamide (**16**)

Guanidine free base solution was prepared by mixing guanidine·HCl (100 mg, 1.05 mmol) and NaH (60% w/w oil dispersion, 42 mg, 1.05 mmol) in dry DMF (500 μL). Intermediate **15** (50 mg, 87 μmol, 1 equiv), DABCO (20 mg, 174 μM, 2 equiv), and guanidine free base solution (168 μL, 349 μmol, 4 equiv) were added to dry DMF (300 μL). The reaction mixture was stirred overnight and monitored by LCMS. The reaction mixture was crashed out in water (10 mL) and washed with diethyl ether (3 × 10 mL). The resulting solid was HPLC prep purified (0–100% Buffer B over 60 min) and lyophilized to give a white powder (38 mg, 76%). Solvent system: Buffer A, 95:5:0.1 H_2_O/ACN/TFA; Buffer B, 95:5:0.1 ACN/H_2_O/TFA.

^1^H NMR (500 MHz, MeOD) δ 8.52 (d, *J* = 7.5 Hz, 1H), 8.19–8.12 (m, 2H), 7.31–7.23 (m, 2H), 5.50 (d, *J* = 2.1 Hz, 2H), 4.01 (bs, 1H), 3.90 (m, 2H), 2.98–2.79 (m, 2H), 1.86–1.71 (m, 8H), 1.17–1.08 (m, 1H), 0.65–0.52 (m, 2H), 0.48–0.34 (m, 2H). ^13^C NMR (126 MHz, MeOD) δ 174.5, 166.1, 165.7, 165.6 (d, *J* = 249.2 Hz), 163.43, 163.14, 157.89, 156.74, 130.05 (d, *J* = 8.8 Hz), 125.0 (d, *J* = Hz), 119.1, 117.1 (d, *J* = 22.4 Hz), 55.8, 54.2, 53.9, 47.9, 43.3, 43.0, 28.8, 28.7, 16.5, 4.3, 4.2, 4.0. HRMS (ESI): calculated for C_25_H_34_FN_14_O_2_ [M+H]^+^ 581.2968, found 581.2969.

#### 3-((4-Amino-6-(((1s,4s)-4-(2-(5-(4-fluorophenyl)-2*H*-tetrazol-2-yl)acetamido)cyclohexyl)amino)-1,3,5-triazin-2-yl)amino)-3-cyclopropylpropanamide (**17**)

Intermediate **15** (25 mg, 45 μmol, 1 equiv) and DABCO (5 mg, 45 μmol, 1 equiv) were dissolved in 7 M ammonia in MeOH (1 mL) before being warmed to 65 °C overnight. The solvent was removed, and the oil was redissolved in 7 M ammonia in MeOH (1 mL) and warmed to 65 °C overnight. This process was repeated until more than half of the starting material was consumed (2:1 product to starting material). The organic solvent was removed, and the resulting solid was HPLC prep purified (0–100% Buffer B over 60 min) and lyophilized to give a white powder (10 mg, 41%). Solvent system: Buffer A, 95:5:0.1 H_2_O/ACN/TFA; Buffer B, 95:5:0.1 ACN/H_2_O/TFA.

^1^H NMR (500 MHz, MeOD) δ 8.48 (d, *J* = 7.0 Hz, 1H), 8.19–8.12 (m, 2H), 7.30–7.23 (m, 2H), 5.50 (s, 2H), 4.08–3.94 (m, 1H), 3.90 (s, 1H), 3.87–3.75 (m, 1H), 2.66–2.53 (m, 2H), 1.88–1.70 (m, 8H), 1.13–1.01 (m, 1H), 0.65–0.55 (m, 1H), 0.55–0.48 (m, 1H), 0.47–0.39 (m, 1H), 0.39–0.30 (m, 1H). ^13^C NMR (126 MHz, MeOD) δ 176.0, 175.9, 166.6, 166.1, 165.7, 164.6, 130.1 (d, *J* = 8.7 Hz), 125.0 (d, *J* = 3.4 Hz), 117.1 (d, *J* = 22.4 Hz), 55.8, 54.4, 41.2, 28.8, 16.6, 4.3, 3.9, 3.7. HRMS (ESI): calculated for C_24_H_32_FN_12_O_2_ [M+H]^+^ 539.2750, found 539.2753.

### Antibacterial Activity Assays

Determination of MIC and synergistic activity was carried out according to Clinical and Laboratory Standards Institute (CLSI) guidelines. The strains used in this study are as follows: *E. coli* ATCC 25922, *K. pneumoniae* ATCC 13883, *A. baumannii* ATCC 9955, and *P. aeruginosa* ATCC 27853. *E. coli* BW28113 was provided by Dennis Doorduijn, Microbiology UMC, NLD; *MRSA* USA 300 was provided by Antoni Hendrick, UMCU, NLD; *MSSA* 29213 was provided by Linda Quarles van Ufford, Utrecht, NLD.

### MIC Assays

A single colony of the test bacteria was inoculated in tryptic soy broth (TSB) and incubated at 37 °C with shaking. The bacterial cells were grown to an OD_600_ of 0.5 and then diluted with Mueller–Hinton broth (MHB) to a final concentration of 10^6^ CFU/mL. Compounds stocks were prepared in MHB as a 2× final concentration. The compounds were serially diluted with MHB in polypropylene 96-well plates (50 μL in each well). The bottom row of each plate was used for positive (50 μL MHB/50 μL bacteria) and negative (100 μL MHB) controls. The bacterial stock was added to the microplate (50 μL to each well, final volume 100 μL). The microplates were incubated at 37 °C for 16–20 h and inspected for bacterial growth. The MIC was defined as the lowest concentration of the compound that prevented visible growth of the bacteria.

### Synergy Assays

Test compounds were diluted to 4× the final concentration needed using MHB. They were then serially diluted with MHB, the maximum concentration being equal to their MIC (25 μL in each well). Rifampicin was diluted to 4× the final concentration needed for each combination and added to the test compounds (25 μL). The bacteria were inoculated and prepared as described above before being added to the plate (50 μL of suspension added, final volume: 100 μL). The plates were incubated at 37 °C for 16–20 h, after which the optical density of each well was read by a Tecan Spark plate reader at 600 nm. The FICI of each combination was established, and a value of <0.5 indicates synergy. The combination of compound and rifampicin that gave the lowest value was reported according to the following equation:
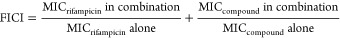


### Membrane Permeabilization Assay

This assay was performed based on a protocol adapted from those previously described in literature.^24,25^ Bacteria were grown overnight at 37 °C in LB, diluted 50× in Lysogeny Broth (LB), and then re-grown to an OD_600_ of 0.5. The bacterial suspension was centrifuged for 10 min at 1000*g*. The bacterial pellet was then resuspended in 5 mM HEPES buffer supplemented with 20 mM glucose to a final OD_600_ concentration of 1.0. The test compounds were serially diluted (25 μL) in triplicate in a black, 1/2 area clear-bottom 96-well plate. Colistin (final concentration 100 μg/mL) was used as the positive control and DMSO (25 μL) was used as the negative control. To ensure no interactions between the compounds and NPN occur, three wells were filled as an additional control with 25 μL of the highest concentration of compound, NPN, and buffer without the presence of bacteria. A 0.5 mM stock of NPN in acetone was prepared which was further diluted to 12.5× in assay buffer. The NPN solution (25 μL) was added to each well. The 1.0 OD_600_ bacterial stock (50 μL) was then added to all appropriate wells. Wells that were to receive no bacteria received assay buffer instead (50 μL). After 60 min, the plate was measured using a Tecan plate reader with λ_ex_ = 355 ± 20 nm and λ_em_ = 420 ± 20 nm. The fluorescence values obtained were transformed into NPN uptake percentage using the following equation:
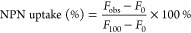
where the observed fluorescence (*F*_obs_) is corrected for background using the negative control (*F*_0_). This value is divided by the positive control corrected for the background (*F*_100_*– F*_0_) and multiplied by 100% to obtain the percentage NPN uptake:

### Hemolysis Assay

Red blood cells from defibrinated sheep blood were obtained from Thermo Fisher. These cells were centrifuged (400*g*, 15 min, 4 °C) and washed five times with phosphate-buffered saline (PBS) containing 0.002% Tween20. The red blood cells were normalized to obtain a positive control read-out of 2.5 at 415 nm to stay within the linear range with the maximum sensitivity. A serial dilution of the compounds (75 μL) was prepared in a 96-well plate, and each compound was assessed in triplicate. Each plate contained 0.1% Triton-X as a positive control (75 μL) and buffer as a negative control (75 μL) in triplicate. The normalized blood cells (75 μL) were added and the plates were incubated at 37 °C for 1 or 18 h while shaking at 500 rpm. A flat-bottom polystyrene plate with buffer (100 μL) in each well was prepared. The plates were centrifuged (800*g*, 5 min), and 25 μL of the supernatant was transferred to the previously prepared plate. The plates were measured using a Tecan plate reader at 415 nm. The values obtained were corrected for background and transformed to a percentage relative to the positive control.

### Rcs Stress Response Assays

The effect of MRL-494 and analogues on bacterial growth and Rcs stress induction was determined using *E. coli* Top10F′ cells harboring the P*rprA*-mNG Rcs reporter construct as previously described.^19^

## Supporting Information Available

Supporting figures and tables, ^1^H and ^13^C NMR spectra, and analytical RP-HPLC traces for final compounds (PDF)

## Notes

Samples of all compounds reported are available from the authors upon request.

## Abbreviations

ACN: acetonitrile
BAM: β-barrel assembly machine
DABCO: 1,4-diazabicyclo[2.2.2]octane
DCM: dichloromethane
DIPEA: *N,N*-diisopropylethylamine
DMF: dimethylformamide
DMSO: dimethyl sulfoxide
EtOH: ethanol
FICI: fractional inhibitory concentration index
HRMS: high-resolution mass spectrometry
IM: inner membrane
MeOH: methanol
MIC: minimum inhibitory concentration
NaH: sodium hydride
NaN_3_: sodium azide
NaOEt: sodium ethoxide
NaOH: sodium hydroxide
NEt_3_: triethylamine
NPN: *N*-phenylnaphthalen-1-amine
NH_3_: ammonia
OM: outer membrane
OMP: outer membrane protein
PMBN: polymyxin B nonapeptide
PMEN: polymyxin E nonapeptide
POTRA: polypeptide transport associated (domains)
Rcs: regulation of capsular polysaccharide synthesis
RP-HPLC: reverse-phase high-performance liquid chromatography
TFA: trifluoroacetic acid
THF: tetrahydrofuran
