# Supplementary material for: Synthesis and Structure–Activity Studies of β-Barrel Assembly Machine Complex Inhibitor MRL-494
Source: ACS Infect Dis. 2022 Nov 1;8(11):2242–52. doi: 10.1021/acsinfecdis.2c00459 (PMC9673140; doi:10.1021/acsinfecdis.2c00459)

Supporting Information for:

Synthesis and structure-activity studies of BAM complex inhibitor MRL-494

Nicola Wade,[a] Charlotte M. J. Wesseling,[a] Paolo Innocenti,[a] Cornelis J. Slingerland,[a] Gregory M. Koningstein,[b] Joen Luirink,[b] and Nathaniel I. Martin*[a]

[a]*Biological Chemistry Group, Institute of Biology Leiden, Leiden University, 2333 BE Leiden, The Netherlands.*

[b]*Department of Molecular Microbiology, Amsterdam Institute of Molecular and Life Sciences (AIMMS), Vrije Universiteit, 1081HV Amsterdam, The Netherlands.*

*n.i.martin@biology.leidenuniv.nl

Table of Contents

S2	....	General information
S3	....	Synthesis
S4	....	Gram-positive MIC results
S4	....	Checkerboard results
S5	....	Synergy plots
S7	....	Hemolysis data
S9	....	Rcs stress response data
S10	....	^{1}H and ^{13}C NMR spectra
S14	....	Analytical RP-HPLC data

General information

HRMS analysis was performed on a Shimadzu Nexera X2 UHPLC system with a Waters Acquity HSS C18 column (2.1 × 100 mm, 1.8 µm) at 30 °C and equipped with a diode array detector. The following solvent system, at a flow rate of 0.5 mL/min, was used: solvent A, 0.1 % formic acid in water; solvent B, 0.1 % formic acid in acetonitrile. Gradient elution was as follows: 95:5 (A/B) for 1 min, 95:5 to 15:85 (A/B) over 6 min, 15:85 to 0:100 (A/B) over 1 min, 0:100 (A/B) for 3 min, then reversion back to 95:5 (A/B) for 3 min. This system was connected to a Shimadzu 9030 QTOF mass spectrometer (ESI ionisation) calibrated internally with Agilent's API-TOF reference mass solution kit (5.0 mM purine, 100.0 mM ammonium trifluoroacetate and 2.5 mM hexakis(1H,1H,3H-tetrafluoropropoxy)phosphazine) diluted to achieve a mass count of 10000.

HPLC analyses were performed on a Shimadzu Prominence-i LC-2030 system with a Dr. Maisch ReproSil Gold 120 C18 column (4.6 × 250 mm, 5 or 10 µm) at 30 °C and equipped with a UV detector monitoring at X and Y nm. The following solvent system, at a flow rate of 1 mL/min, was used: solvent A, 0.1 % TFA in water/acetonitrile 95/5; solvent B, 0.1 % TFA in water/acetonitrile 5/95. Gradient elution was as follows: 95:5 (A/B) for 2 min, 95:5 to 0:100 (A/B) over 13 min, 0:100 (A/B) for 2 min, then reversion back to 95:5 (A/B) over 1 min, 95:5 (A/B) for 2 min.

Preparative HPLC runs were performed on a BESTA-Technik system with a Dr. Maisch Reprosil Gold 120 C18 column (25 × 250 mm, 10 µm) and equipped with a ECOM Flash UV detector monitoring at X nm. The following solvent system, at a flow rate of 12 mL/min, was used: solvent A, 0.1 % TFA in water/acetonitrile 95/5; solvent B, 0.1 % TFA in water/acetonitrile 5/95. Gradient elution was as follows: 95:5 (A/B) for 2 min, 95:5 to 0:100 (A/B) over 13 min, 0:100 (A/B) for 2 min, then reversion back to 95:5 (A/B) over 1 min, 95:5 (A/B) for 2 min.

Building block synthesis

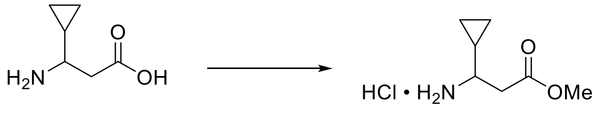

S1

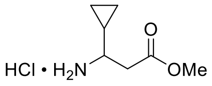

S1

*(±)-Methyl 3-amino-3-cyclopropylpropanoate **(S1)**.* (±)-3-amino-3-cyclopropyl-propionic acid (500 mg, 3.87 mmol, 1 eq) was dissolved in methanol (15 mL) and cooled to 0 °C. Thionyl chloride (600 µL, 8.25 mmol, 2.1 eq) was added dropwise to the solution and stirred for 3 h before gradually warming to room temperature. The reaction was stirred for a further 18 h and monitored by TLC (99.5/0.5 DCM/NEt_3). When the reaction was complete, the solvent was removed and mixture coevaporated with toluene (3 x 10 mL) to give a white solid (quant). This was used in the next step without further purification.

^{1}H NMR (400 MHz, MeOD) δ 3.74 (s, 3H), 2.93 – 2.77 (m, 3H), 1.13 – 1.02 (m, 1H), 0.76 – 0.64 (m, 2H), 0.57 – 0.50 (m, 1H), 0.44 – 0.37 (m, 1H). ^{13}C NMR (101 MHz, MeOD) δ 172.2, 55.1, 52.7, 37.9, 14.5, 4.8, 4.4. HRMS (ESI): calculated for $C_7H_{14}NO_2$ [M+H]+ 144.1019, found 144.1020.

Table S1. Gram-positive bacteria MIC results.

Strain	**1**	**13**	**16**	**17**
MSSA 29213	8	64	128	>128
MRSA USA 300	8	64	128	>128

Table S2. Results of **13** checkerboard assays in combination with rifampicin.

Strain	MIC (μg/mL)				FICI
	**13** alone	**13** in combination	Rifampicin alone	Rifampicin in combination	
E. coli ATCC 25922	>128	32	2	0.125	≤0.188
E. coli BW25113	64	32	4	0.125	0.281
K. pneumoniae ATCC 13883	>128	16	8	1	≤0.125
A. baumannii ATCC 9955	>128	16	1	0.125	≤0.188
P. Aeruginosa ATCC 27853	>128	-	16	-	-

Table S3. Results of **16** checkerboard assays in combination with rifampicin.

Strain	MIC (μg/mL)				FICI
	**16** alone	**16** in combination	Rifampicin alone	Rifampicin in combination	
E. coli ATCC 25922	128	16	2	0.125	0.188
E. coli BW25113	128	16	4	0.25	0.186
K. pneumoniae ATCC 13883	>128	16	8	1	≤0.125
A. baumannii ATCC 9955	>128	16	1	0.125	≤0.188
P. Aeruginosa ATCC 27853	128	-	16	-	-

Table S4. Results of **17** checkerboard assays in combination with rifampicin.

Strain	MIC (μg/mL)				FICI
	**17** alone	**17** in combination	Rifampicin alone	Rifampicin in combination	
E. coli ATCC 25922	>128	-	2	-	-
E. coli BW25113	>128	-	4	-	-
K. pneumoniae ATCC 13883	>128	-	8	-	-
A. baumannii ATCC 9955	>128	-	1	-	-
P. Aeruginosa ATCC 27853	>128	-	16	-	-

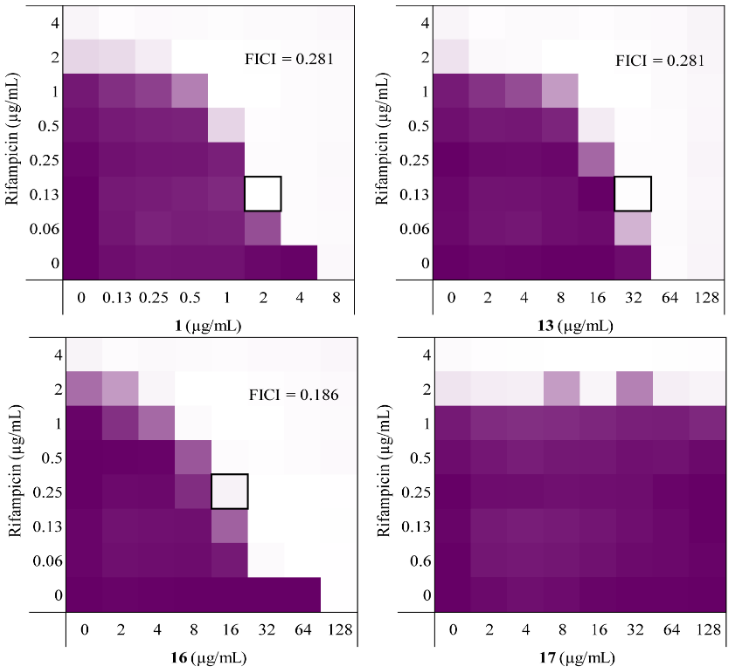

Figure S1. Checkerboard assay results for MRL-494 (**1**) and analogues (**13**, **16**, and **17**) in combination with rifampicin against *E. coli* BW25113. The combination of test compound and rifampicin which resulted in the lowest FICI is indicated by a black box. The mean optical density of the bacterial growth (OD600) is shown as a colour gradient, with purple signifying maximum bacterial growth and white as no growth.

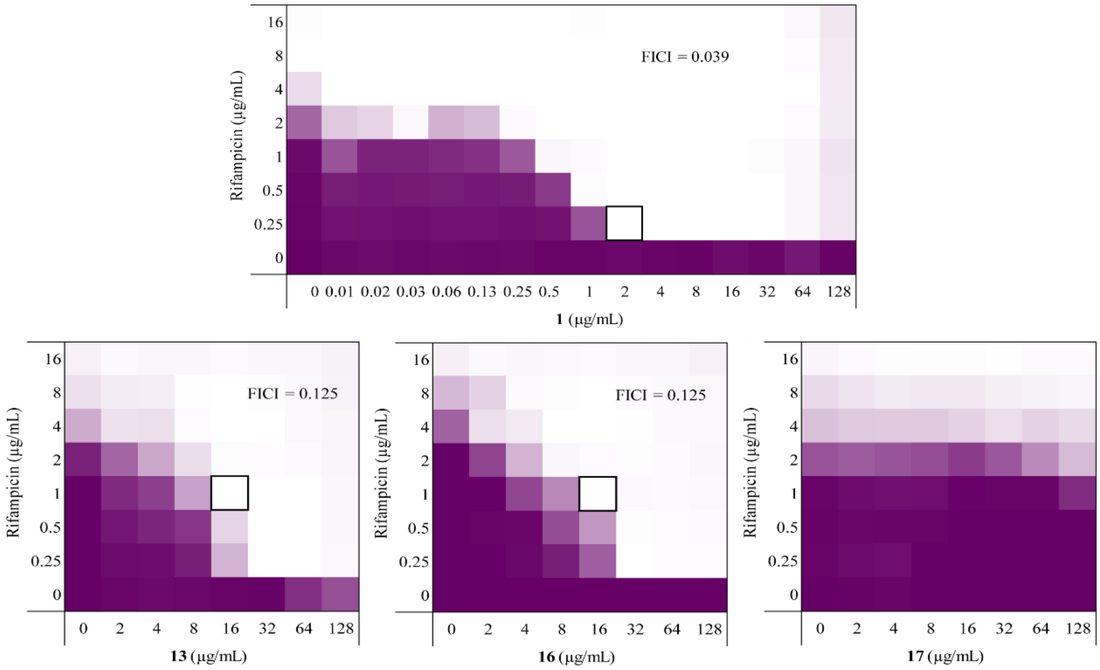

Figure S2. Checkerboard assay results for MRL-494 (**1**) and analogues (**13**, **16**, and **17**) in combination with rifampicin against *K. pneumoniae* ATCC 13883. The combination of test compound and rifampicin which resulted in the lowest FICI is indicated by a black box. The mean optical density of the bacterial growth (OD600) is shown as a colour gradient, with purple signifying maximum bacterial growth and white as no growth.

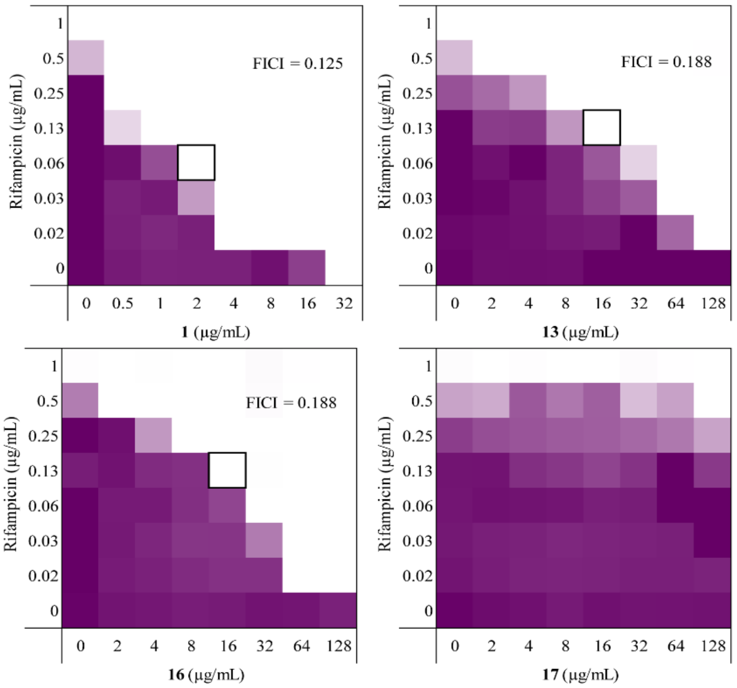

Figure S3. Checkerboard assay results for MRL-494 (**1**) and analogues (**13**, **16**, and **17**) in combination with rifampicin against *A. baumannii* ATCC 9955. The combination of test compound and rifampicin which resulted in the lowest FICI is indicated by a black box. The mean optical density of the bacterial growth (OD600) is shown as a colour gradient, with purple signifying maximum bacterial growth and white as no growth.

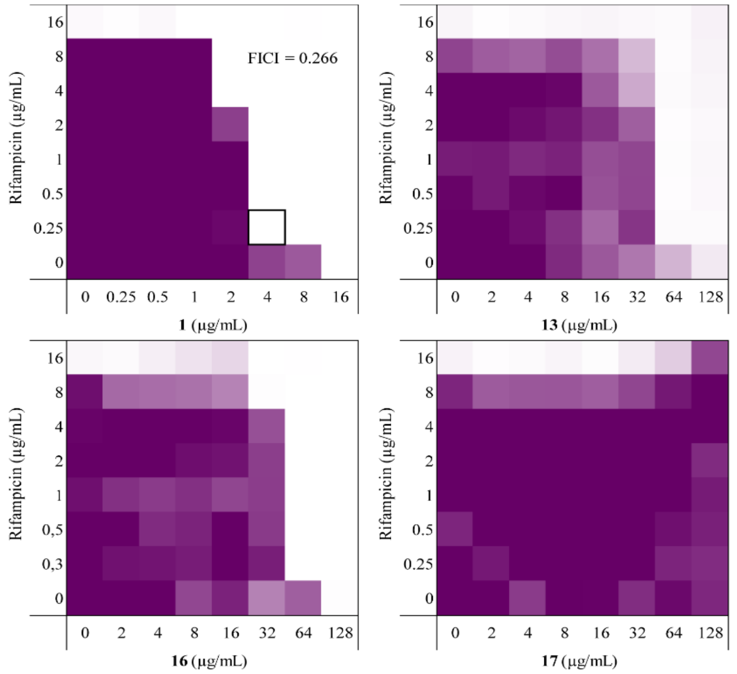

Figure S4. Checkerboard assay results for MRL-494 (**1**) and analogues (**13**, **16**, and **17**) in combination with rifampicin against *P. aeruginosa* ATCC 27853. The combination of test compound and rifampicin which resulted in the lowest FICI is indicated by a black box. The mean optical density of the bacterial growth (OD600) is shown as a colour gradient, with purple signifying maximum bacterial growth and white as no growth.

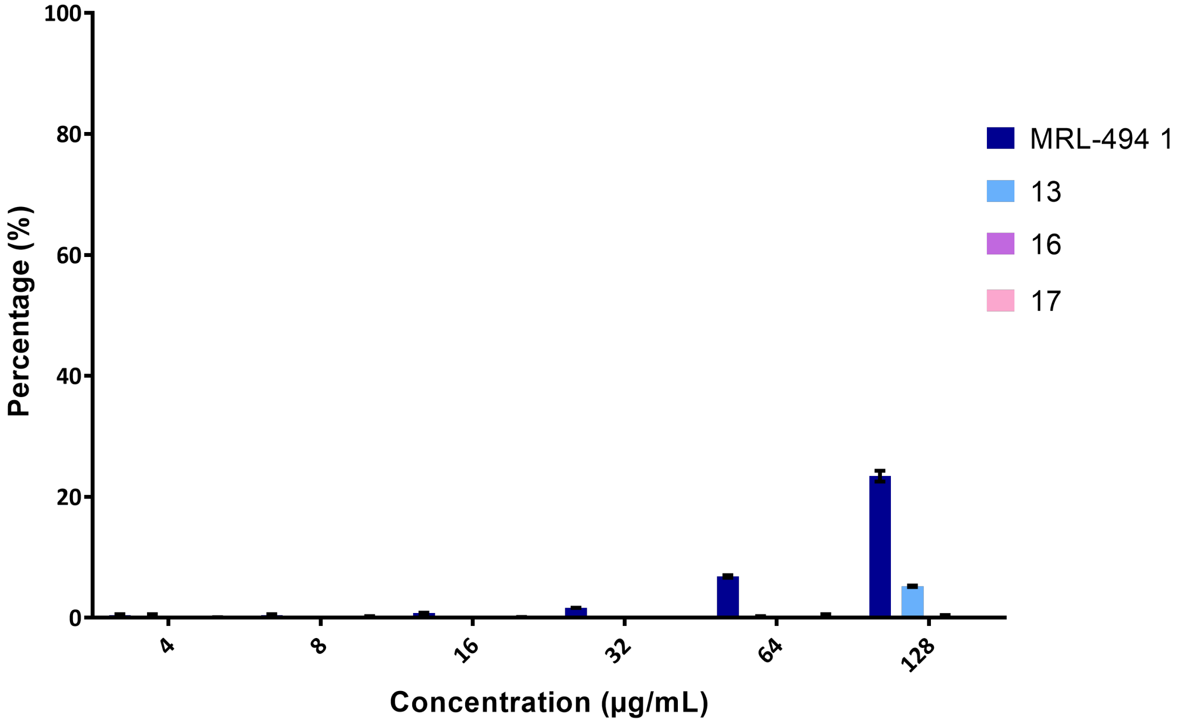

Figure S5. Hemolytic activity of all test compounds after 18 hours of incubation. A description of the hemolysis assay is found in the materials and methods. Error bars are calculated based on n=3 technical replicates.

Table S5. Hemolysis data points

	Concentration (µg/mL)					
	128	64	32	16	8	4
Compound	% Hemolysis					
MRL-494 **1**	23.4	6.8	1.6	0.8	0.4	0.4
**13**	5.2	0.2	-0.3	-0.4	-0.2	0.3
**16**	0.3	-0.4	-0.5	-0.4	-0.3	-0.3
**17**	0.0	0.2	-0.1	0.0	0.1	0.0

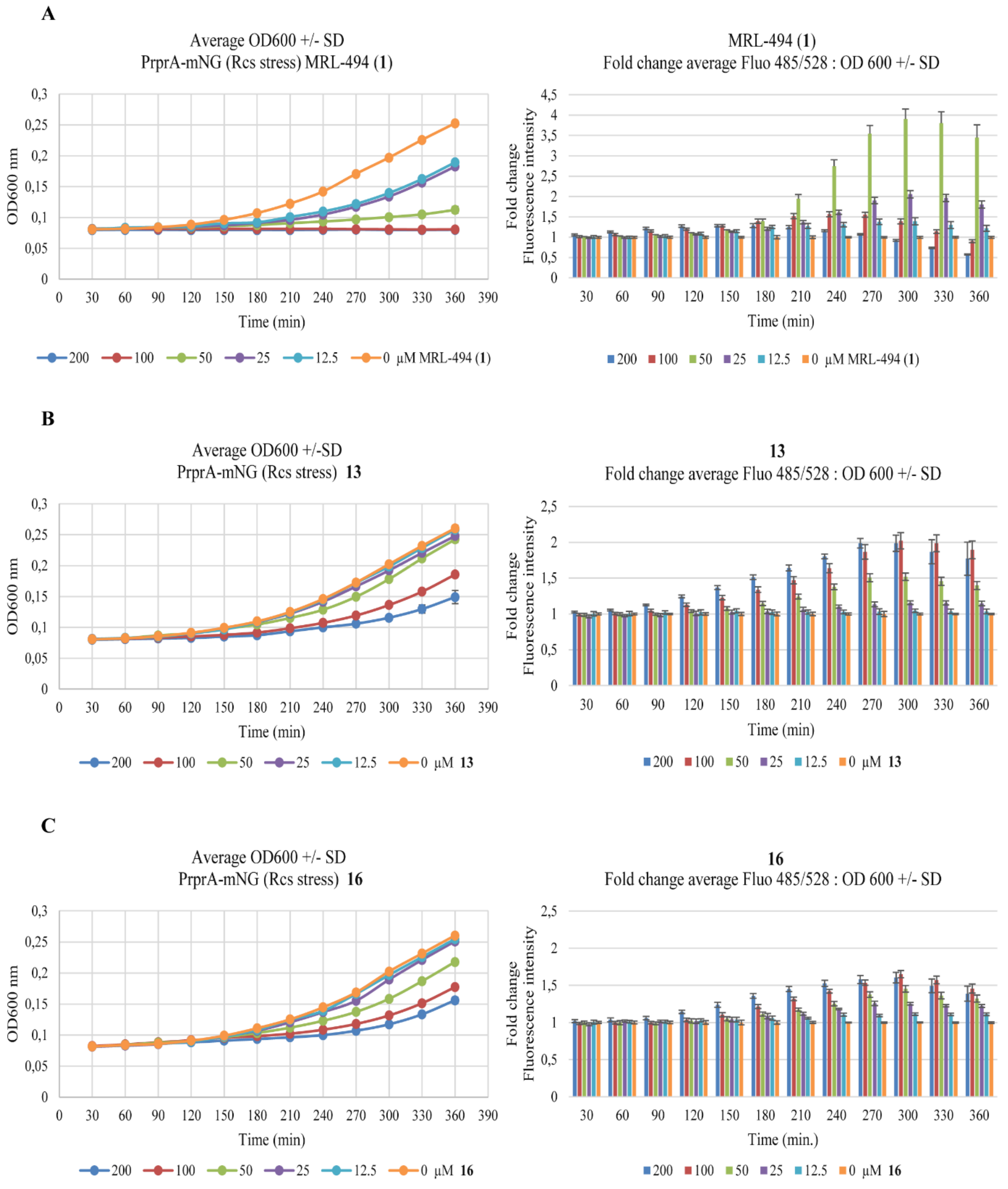

Figure S6. Real-time monitoring of bacterial growth and Rcs stress activation in response to MRL-494 **1** and analogues (**13** and **16**). *E. coli* TOP10F' cells, harboring the PrprA-mNG reporter construct, were grown in a 96-well plate and exposed to the compounds at the indicated concentration at timepoint 0. Growth (OD_{600}) and mNG fluorescence were measured in time. Fluorescence was corrected for growth (OD_{600}) and plotted as fold-change of signal compared to untreated cells (set to 1). Error bars represent the standard deviation of triplicate technical replicates.

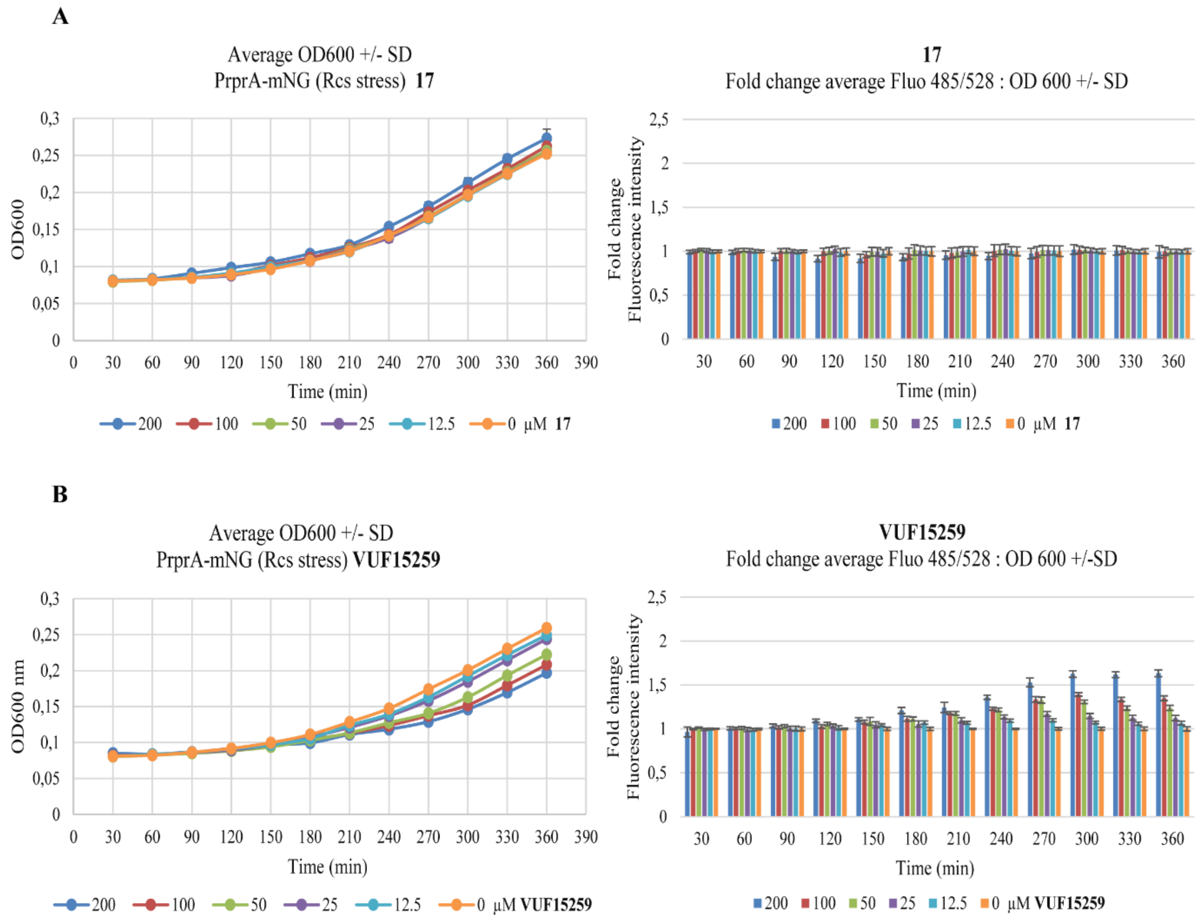

Figure S7. Real-time monitoring of bacterial growth and Rcs stress activation in response to MRL-494 analogue **17** and known BAM complex inhibitor VUF15259 **3**. *E. coli* TOP10F' cells, harboring the PrprA-mNG reporter construct, were grown in a 96-well plate and exposed to the compounds at the indicated concentration at timepoint 0. Growth (OD_{600}) and mNG fluorescence were measured in time. Fluorescence was corrected for growth (OD_{600}) and plotted as fold-change of signal compared to untreated cells (set to 1). Error bars represent the standard deviation of triplicate technical replicates.

^{1}H and ^{13}C NMR spectra for MRL-494 (**1**)

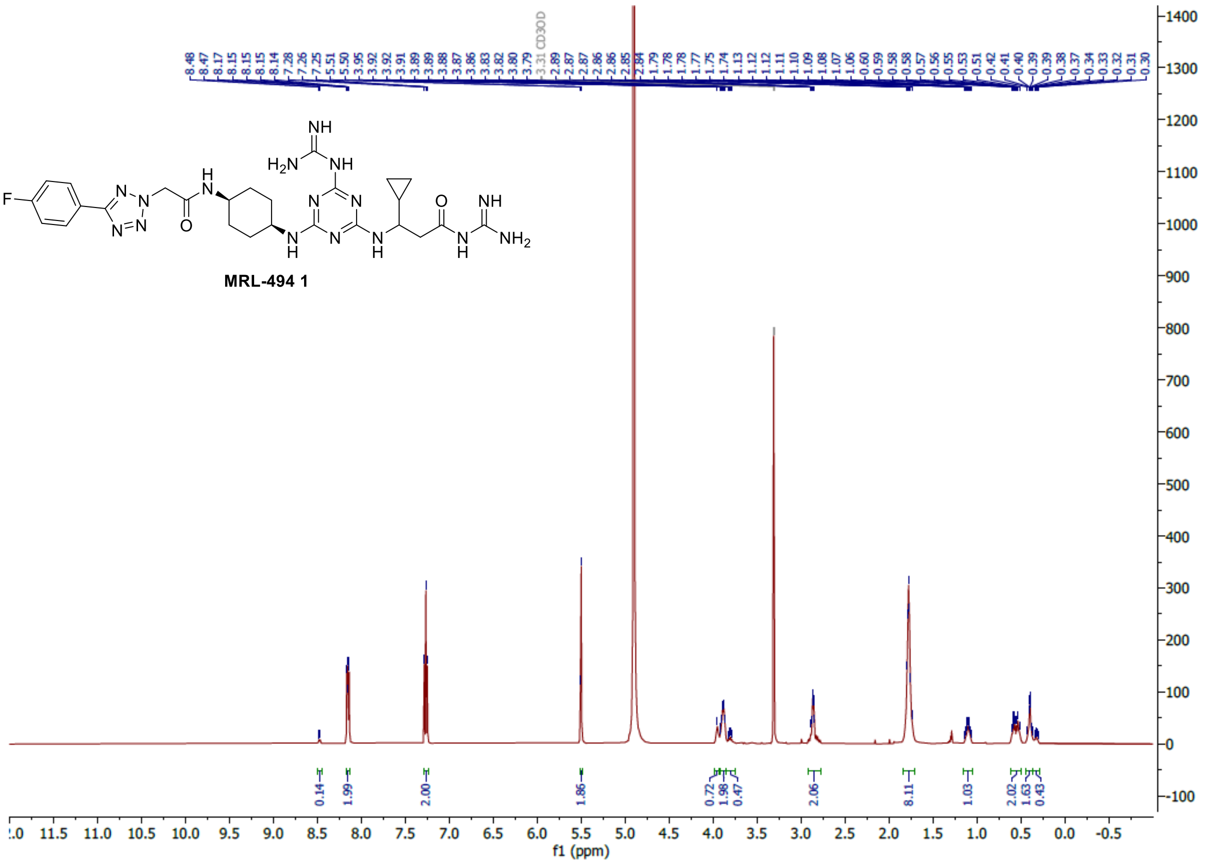

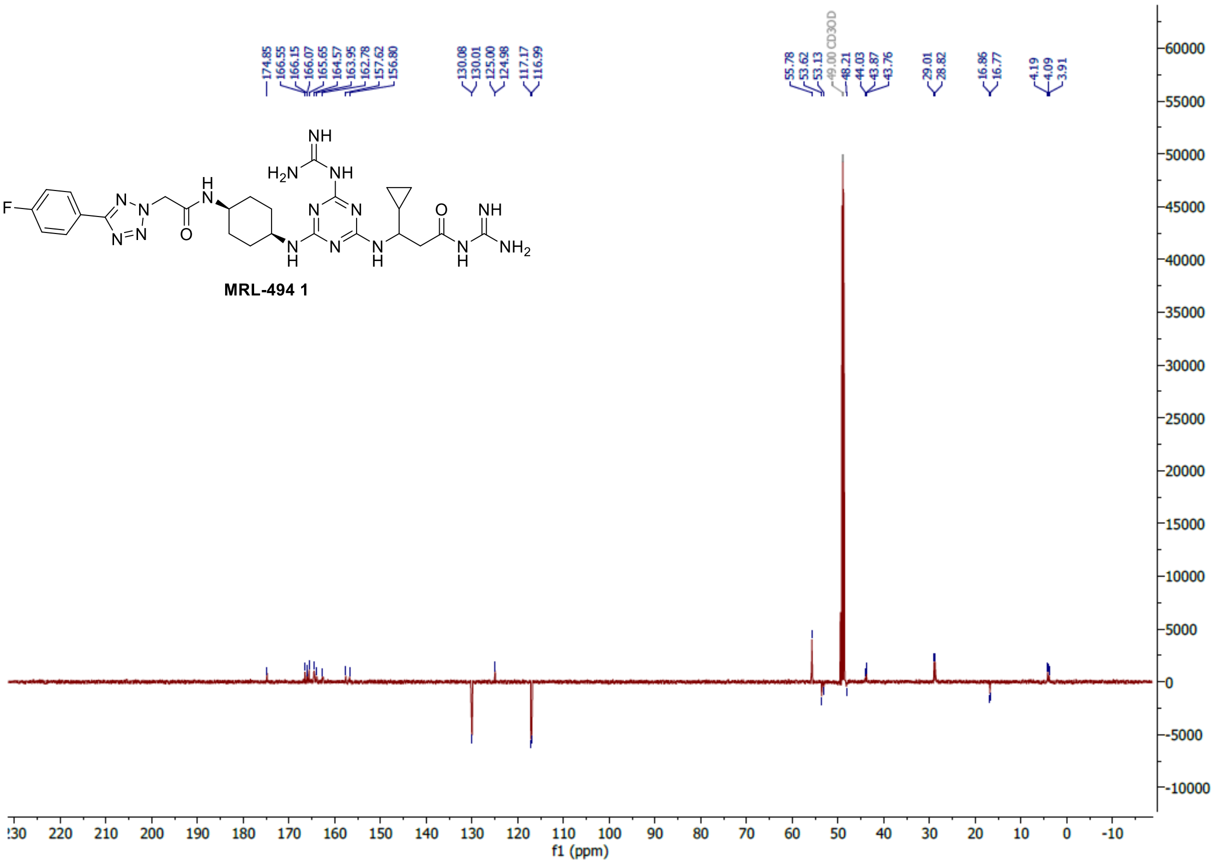

^{1}H and ^{13}C NMR spectra for compound **13**

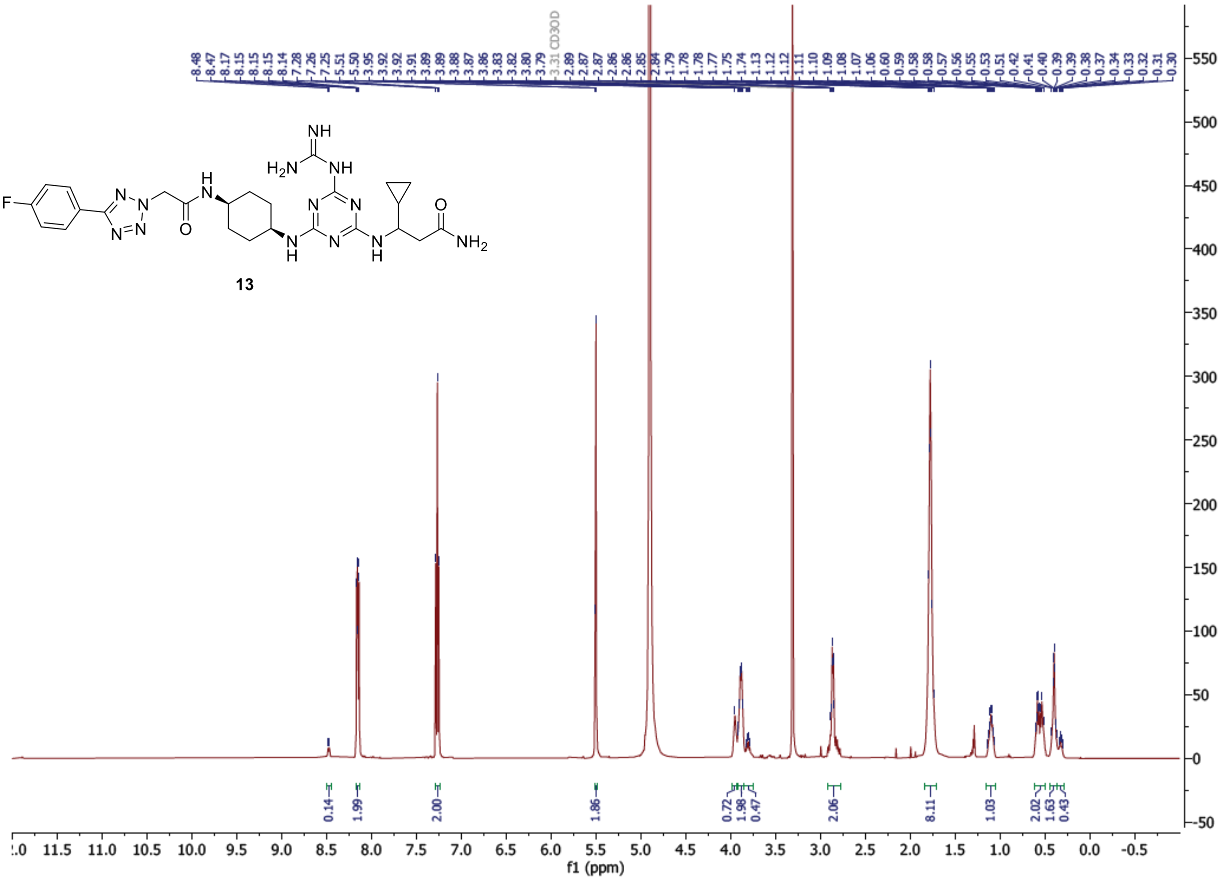

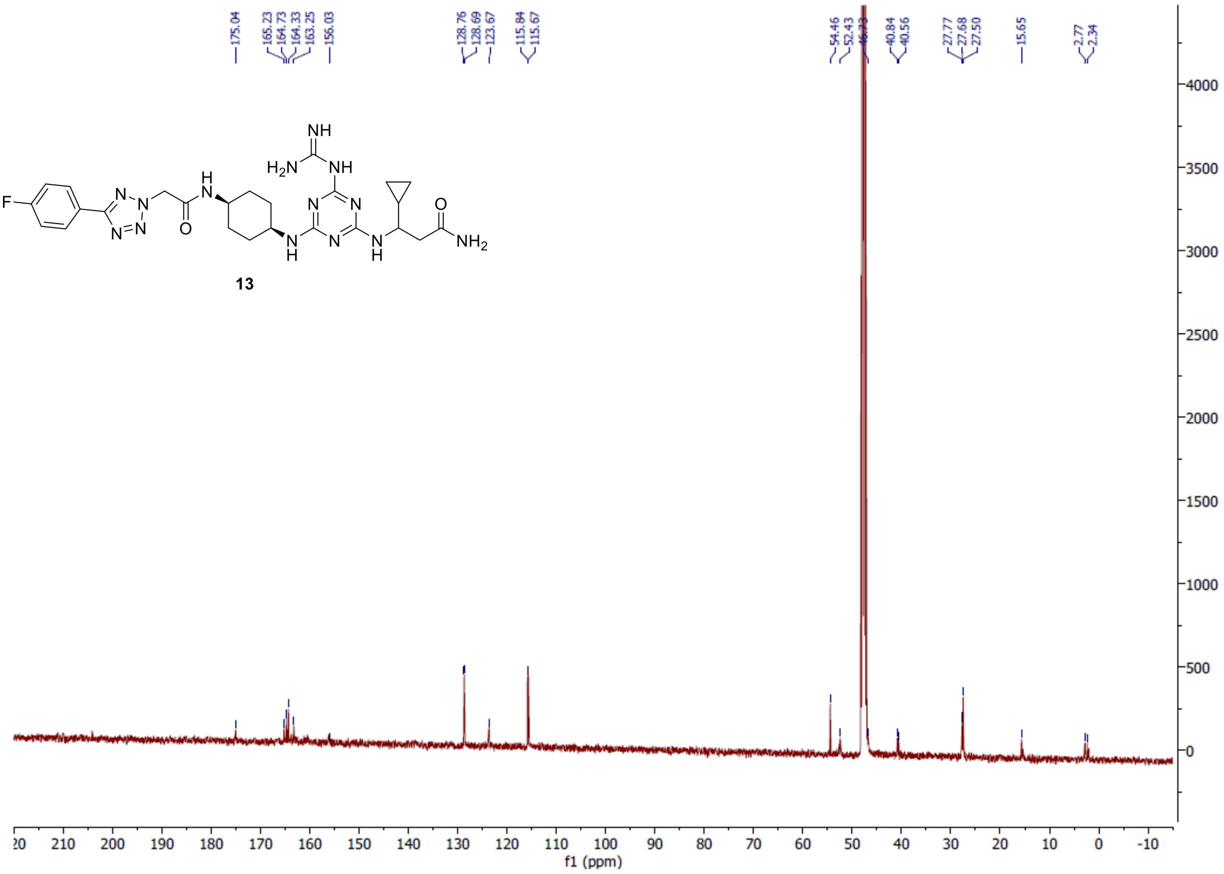

^{1}H and ^{13}C NMR spectra for compound **16**

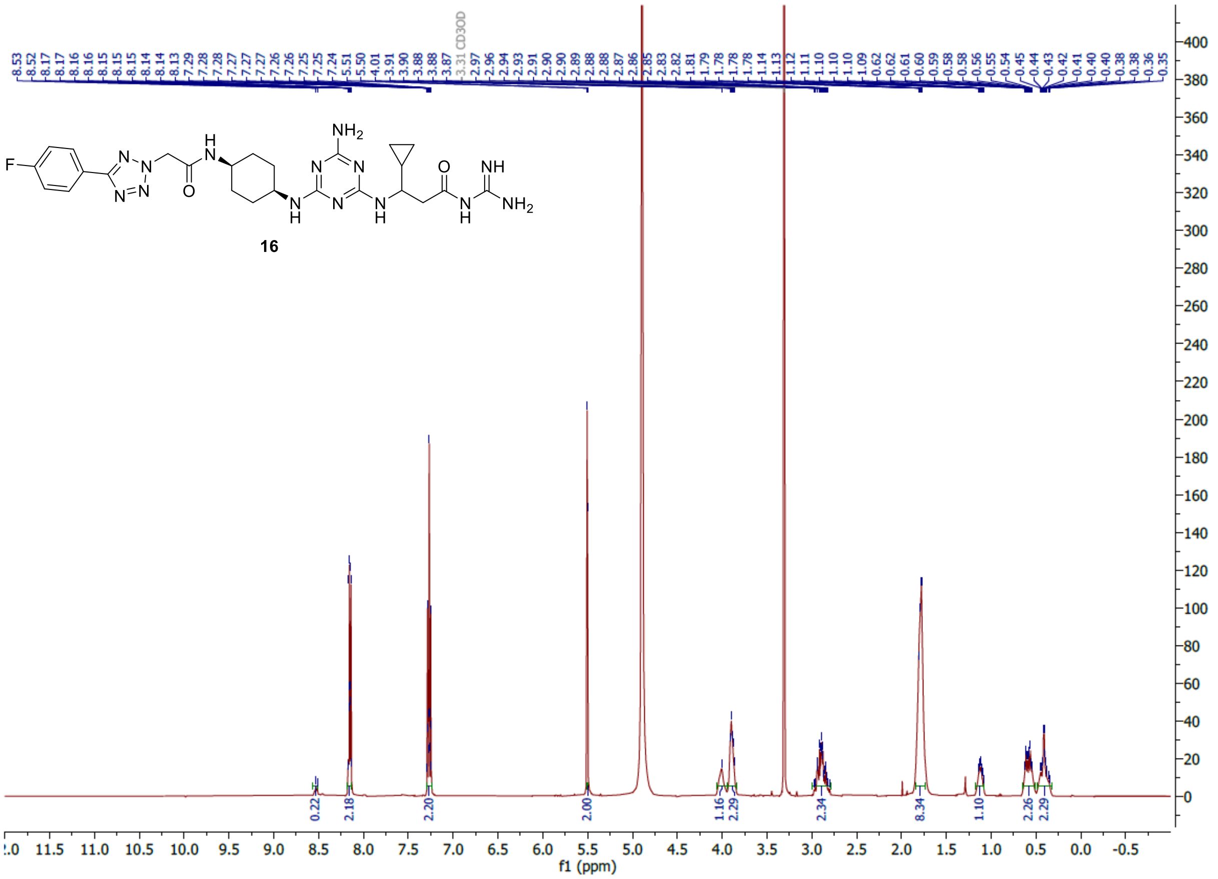

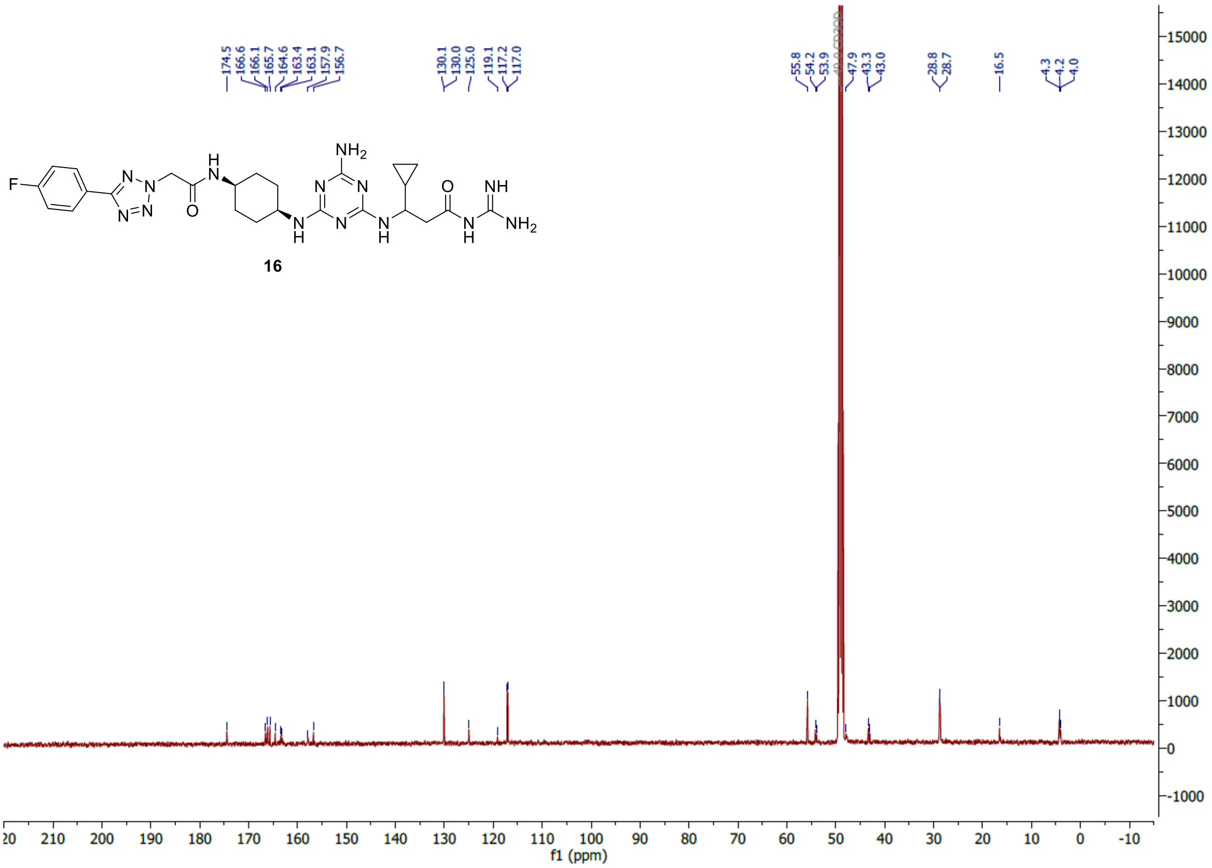

^{1}H and ^{13}C NMR spectra for compound **17**

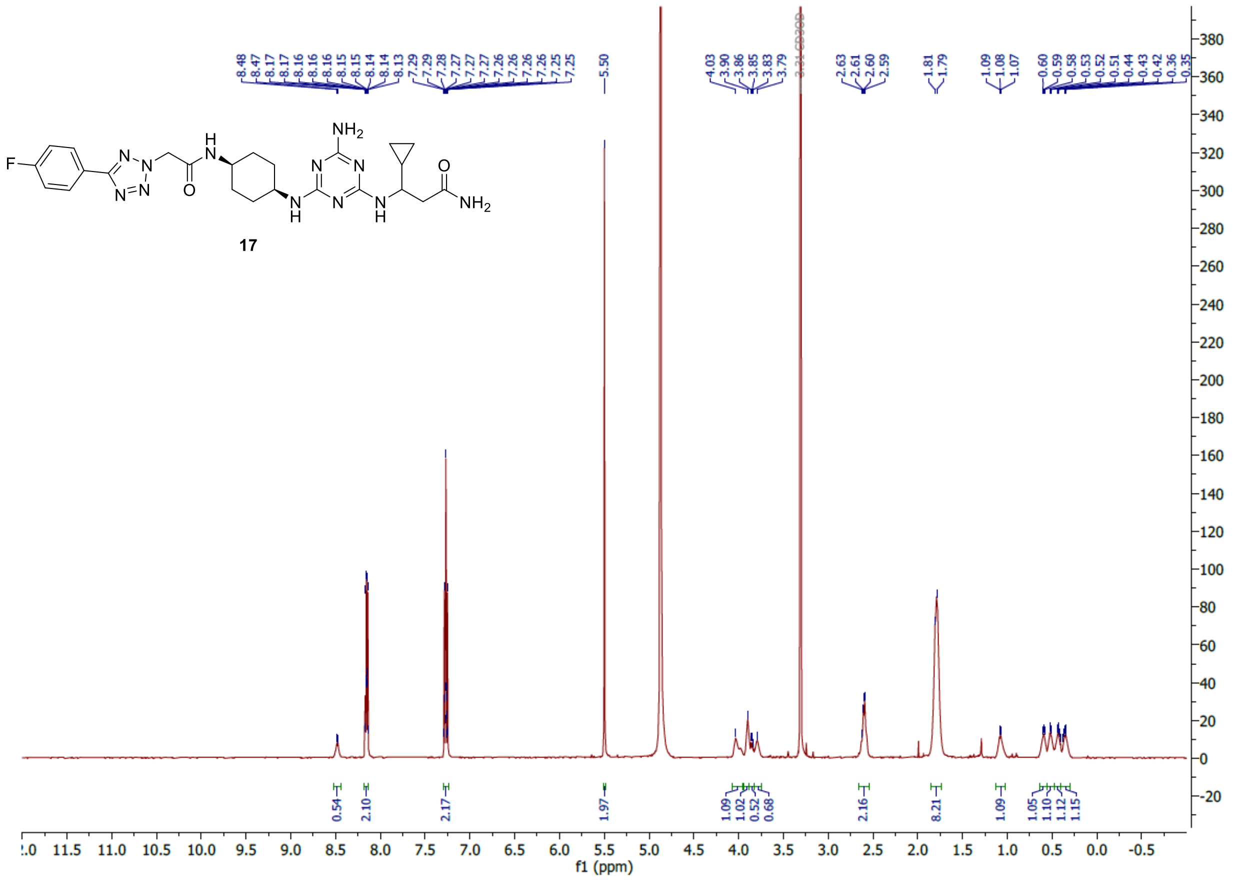

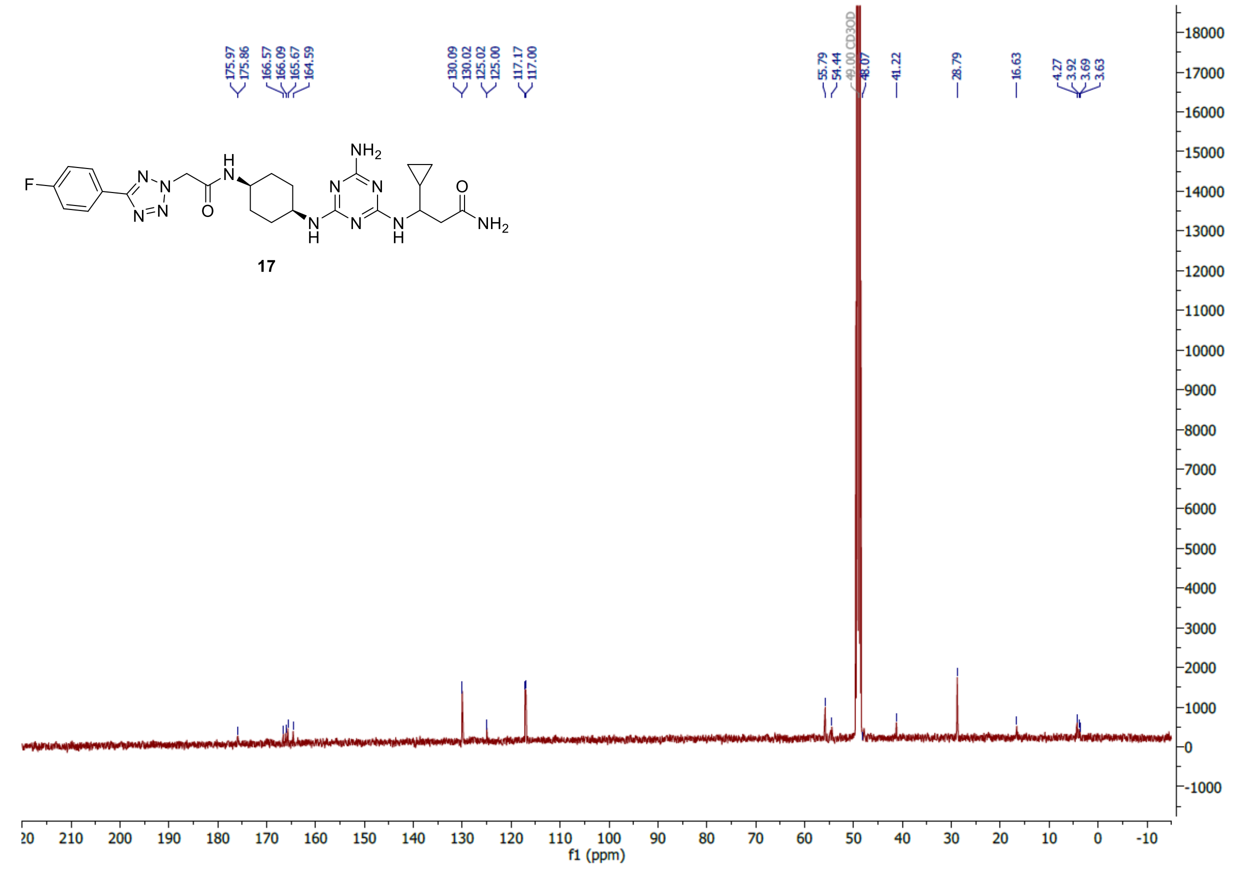

Analytical RP-HPLC data for compound MRL-494 (**1**)

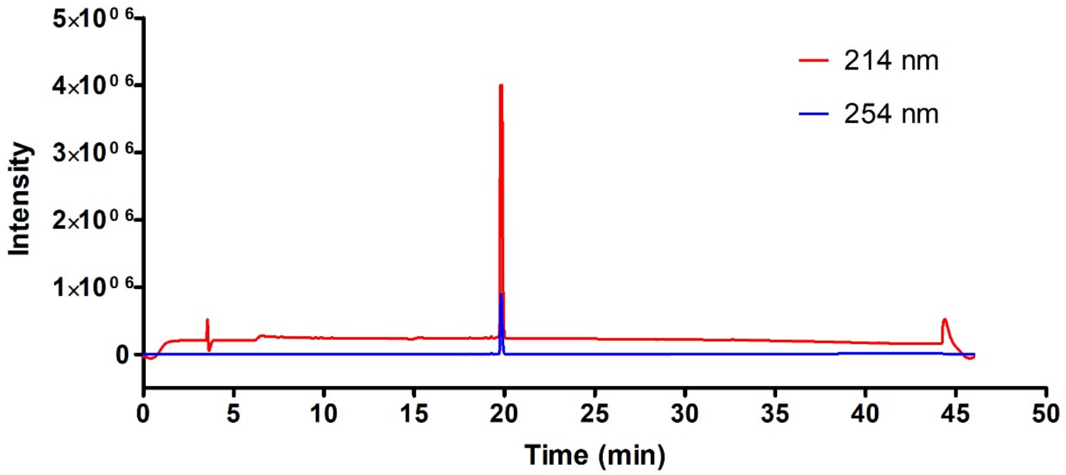

Analytical RP-HPLC data for compound **13**

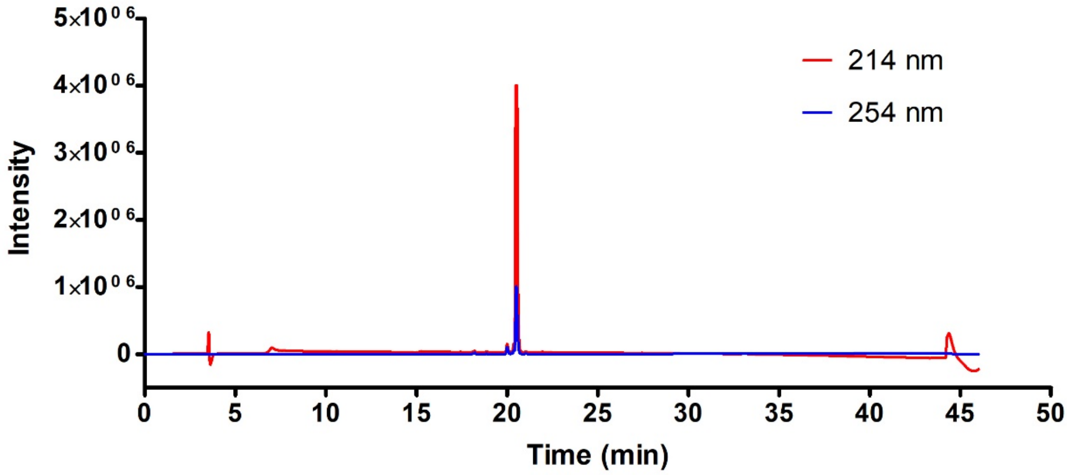

Analytical RP-HPLC data for compound **16**

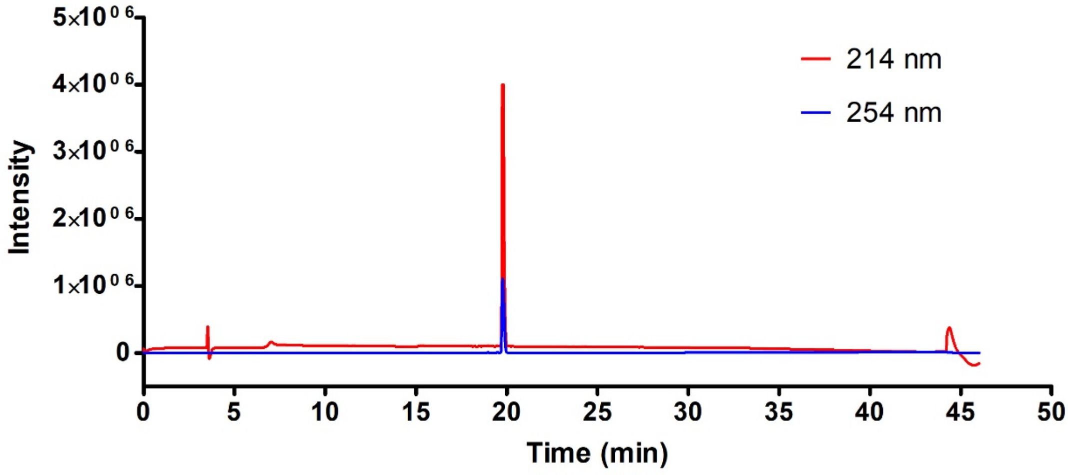

Analytical RP-HPLC data for compound **17**

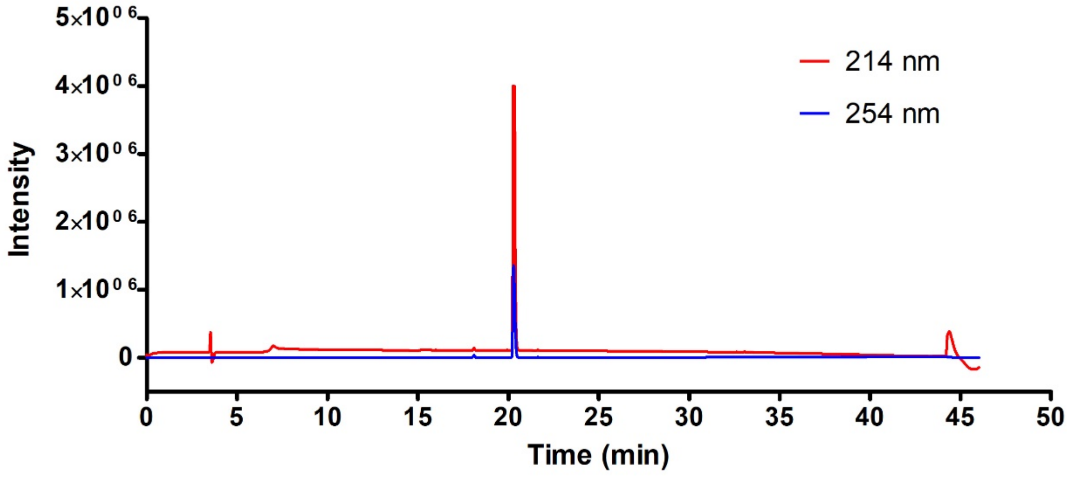

Supplement: Supplementary file 1 — id2c00459_si_001.pdf [file id2c00459_si_001.pdf]
